# Depression among Turkish and Moroccan immigrant populations in Northwestern Europe: a systematic review of prevalence and correlates

**DOI:** 10.1186/s12888-023-04819-4

**Published:** 2023-06-05

**Authors:** Gabriela A. Sempértegui, Christos Baliatsas, Jeroen W. Knipscheer, Marrie H. J. Bekker

**Affiliations:** 1 Tranzo, Tilburg University, Tilburg, The Netherlands; 2 GGz Breburg, Tilburg, The Netherlands; 3 Netherlands Institute for Health Services Research (NIVEL), Otterstraat 118-124, 3513 CR Utrecht, The Netherlands; 4 Arq Psychotrauma Expert Group, Diemen, The Netherlands; 5 Department of Clinical Psychology, Utrecht University, Utrecht, The Netherlands; 6 Department of Clinical, Neuro- and Developmental Psychology, VU University, Amsterdam, The Netherlands

**Keywords:** Depression, Prevalence, Risk factors, Morocco/ethnology, Turkey/ethnology, Europe, Systematic review

## Abstract

**Background:**

This systematic review aimed to synthesize the prevalence and correlates of depressive disorders and symptoms of Turkish and Moroccan immigrant populations in Northwestern Europe, formulating evidence-informed recommendations for clinical practice.

**Methods:**

We conducted a systematic search in PsycINFO, MEDLINE, Science Direct, Web of Knowledge, and Cochrane databases for records up to March 2021. Peer-reviewed studies on adult populations that included instruments assessing prevalence and/or correlates of depression in Turkish and Moroccan immigrant populations met inclusion criteria and were assessed in terms of methodological quality. The review followed the relevant sections of the Preferred Reporting Items for Systematic Reviews and Meta-analyses reporting (PRISMA) guideline.

**Results:**

We identified 51 relevant studies of observational design. Prevalence of depression was consistently higher among people who had an immigrant background, compared to those who did not. This difference seemed to be more pronounced for Turkish immigrants (especially older adults, women, and outpatients with psychosomatic complaints). Ethnicity and ethnic discrimination were identified as salient, positive, independent correlates of depressive psychopathology. Acculturation strategy (high maintenance) was related to higher depressive psychopathology in Turkish groups, while religiousness appeared protective in Moroccan groups. Current research gaps concern psychological correlates, second- and third-generation populations, and sexual and gender minorities.

**Conclusion:**

Compared to native-born populations, Turkish immigrants consistently showed the highest prevalence of depressive disorder, while Moroccan immigrants showed similar to rather moderately elevated rates. Ethnic discrimination and acculturation were more often related to depressive symptomatology than socio-demographic correlates. Ethnicity seems to be a salient, independent correlate of depression among Turkish and Moroccan immigrant populations in Northwestern Europe.

**Supplementary Information:**

The online version contains supplementary material available at 10.1186/s12888-023-04819-4.

## Background

Depression is a global public health priority, associated with chronic physical disorders, elevated risk of early death, and serious functional impairments [1]. While the "healthy immigrant effect" is more prominent in the US, Australia, and Canada [2, 3], in Europe there is growing evidence that immigrant populations are vulnerable to developing mental disorders [4, 5] showing for instance that non-EU immigrants are at increased risk of depression [6, 7].

Turkish and Moroccan communities are among the largest immigrant populations in Europe [8, 9], largely represented in countries such as France, Spain, Germany, and The Netherlands [10–12]. Although some estimations suggest that these two groups have among the highest levels of depressive symptoms in Western Europe [6], a clear overview of the prevalence rates is currently lacking. Hence, the first aim of this review is to synthesize and to examine the available literature and its quality on prevalence of depressive disorders and symptoms of Turkish and Moroccan immigrant populations in Northwestern European countries.

Furthermore, evidence-based therapies for depression, such as pharmacotherapy, Cognitive Behavioral Therapy, and Interpersonal Psychotherapy have barely been investigated in Turkish and Moroccan immigrant groups [13], with the little existing evidence suggesting low effectiveness in treating depression in Turkish migrant populations [14–16]. Moreover, Turkish and Moroccan patients in secondary care tend to receive less intense psychological treatment and to drop out more often of treatment than the native-born in the Netherlands, partly related to gender, age, and illness characteristics [17]. The second aim of the review is to describe the factors that are associated to depressive disorders and symptoms (defined in the present paper as “correlates”) in these groups. Gaining more insight into the correlates of depressive disorders and symptoms could contribute to a better understanding of what aspects are of importance in order to improve treatment effectiveness for depression for these groups.

### Intersectional approach to the correlates of mental health of immigrant populations

In recent years, the public concern in Europe regarding the impact of social problems on mental health of Turkish and Moroccan immigrant populations and vice versa has increased. This concern has certainly been influenced by the migration crisis in Europe since 2015, in which the threat of public stigmatization of refugees has been apparent [18].

A growing body of research has been focusing on socio-demographic factors (e.g., lower socioeconomic status (SES), female gender, single marital status) of immigrant populations as determinants of higher levels of depression [19, 20]. Psychological characteristics like external locus of control and fatalism beliefs have also been linked to depression among immigrants [21]. A number of studies have also examined ethnocultural factors following the narrow definition of culture (including language or traditions, geographic origin, ethnicity, race [22], as well as the broader definition considering social and political processes faced by ethnocultural groups [23, 24]. Among non-EU immigrants, belonging to the first generation, socio-economic marginalization, and ethnic discrimination have been associated with higher levels of depressive symptoms [6, 20]. In addition, the political and economic climate of the host country, reflected in aspects such as provided social support, can influence the degree of integration, success, and wellbeing of immigrant populations [8, 25, 26].

Nevertheless, not all groups of non-EU immigrants show the same prevalence rates or share the same correlates of depression, which points out the need to understand how individual factors, such as socio-demographic or psychological characteristics, ethnocultural factors and contextual factors, interact to contribute to mental health necessities of specific immigrant populations. Thus, the intersectionality theory is gaining space in research on mental health disparities and the efforts to achieve equity [27, 28]. The intersectionality theory was introduced by Crenshaw [29] through her analysis of the social position of Afro-American women. In this analysis, the social categories of race/ethnicity and sex/gender were not studied separately but were observed in their complex interplay [28]. Intersectionality also pays attention to the power systems behind every social category (e.g., racism, sexism) and highlights the risk of higher health burden of individuals in disadvantaged social positions [30].

The intersectionality approach was recently used to investigate the association between ethnic discrimination and common mental disorders amongst African American students [31]. Results showed that young African American men growing up in lower SES conditions and African American women with a higher SES background were at higher risk of mental disorders [31], highlighting the need of examining interactions between socio-demographic and ethnocultural factors to understand inequity and tailor mental health care. In the present study, we used the intersectional perspective to evaluate the results and identify the more vulnerable as well as resilient subgroups within these populations as given by the interplay of socio-demographic, psychological, and/or ethnocultural factors.

## Methods

### Concepts and definitions

Following the risk factors typology and identification methods of Jacobi and colleagues [32], the term ‘correlates’ is used in the present review, to refer to the variables or factors that showed a statistically significant association (positive or negative) with depressive symptomatology level or severity, stemming from single-subject, or between-groups, case–control, cross-sectional studies. Only in the case of longitudinal studies we used the terms ‘protective’ or ‘risk factor’.

Following the approach of another recent review on correlates [19] we grouped the identified correlated into psychological characteristics (e.g. personality dimensions, self-esteem), socio-demographic factors (e.g. sex, age, marital status, educational level, SES, disability), and ethnocultural factors including aspects closely related to migration and social context of the target populations (e.g. acculturation and discrimination). Though often considered a socio-demographic factor, in this review we categorize ethnicity among other ethnocultural factors [22]. We also categorized social support as an ethnocultural factors because we considered important to stress that social support happens within a social and cultural context [33].

In the present paper, we referred to first- and second-generation immigrants as “immigrant populations”. We used the term “native” to refer to all citizens born in the country of residence and whose both parents had also been born in the country of residence. The “native” group also included third- and fourth-generation immigrants because none of the included studies made a distinction between first- and second generation and third and fourth generation, due to the fact that the country of birth (of the person and his/her parents) is the most commonly used identifier of migration status in the literature. Furthermore, even though our search strategy was originally designed to retrieve studies from all Europe, we narrowed the focus of the results and conclusions to Northwestern Europe due to the lack of studies addressing prevalence or correlates of depression that were retrieved outside this region.

### Search strategy

We conducted a systematic review of studies following the PRISMA guidelines for systematic reviews [34]. We performed a computer-based search for studies published between 1970 and March 2021, in five major bibliographic databases: PubMed, PsychInfo, Web of Knowledge, Cochrane and Science Direct. The first author performed the primary search and the second author the update searches. The detailed search strategy including all key word combinations is provided in Additional file 1. Only studies published between 2000 and 2021 were retrieved.

### Inclusion and exclusion criteria

The eligibility criteria of the present review are described in detail in a previous publication [13]. In summary, we included full papers in English, Dutch or German that described studies including:

- a) Samples completely or partially consisted of participants 18 years and older.
- b) Turkish and/or Moroccan samples from multiple or one of the European countries with large Turkish or Moroccan immigrant groups, specifically: United Kingdom, the Netherlands, Belgium, France, Spain, Portugal, Germany, Austria, Switzerland, Italy, Finland, Denmark, Norway, and Sweden [9].
- c) Samples exclusively consisted of Turkish or Moroccan immigrant individuals or samples that included Turkish or Moroccan immigrants in a larger, mixed sample of diverse immigrant groups.
- d) Provision of broad information on depressive disorders and symptoms, or relevant to the treatment of depression. Eligible studies included at least one instrument, subscale or measure of depression (objective clinical diagnoses of depression following DSM or ICD, as registered in primary/secondary/specialized care, were considered as well) and/or stated that all or at least the majority (> 50%) of the sample received treatment for depression or had depressive symptoms consistent with the reported instrument or the pertinent DSM or ICD diagnoses. The eligible studies are included in Additional file 2.

### Data synthesis

#### Quality assessment

Two reviewers independently assessed the methodological quality of the eligible studies, following the approach described in Sempértegui et al. [13]. Studies were classified as being of ‘weak’ (WQ), ‘moderate’ (MQ), or ‘strong’ quality (SQ) (see Additional file 3).

#### Correlates

A box-score method was used to quantify the relationships between the identified psychological variables and depressive symptoms. To this purpose, we tabulated each variable and its association (positive, negative or neutral) with depressive symptoms per study. This implied that more than one association could belong to one study, especially regarding multifactorial variables. Using the quality rating of each study, we calculated the average quality ratings of the mentioned relationships per association type (see Table 2) and per variable (mentioned in the text). A ‘missing value’ was defined as a variable that was included in the analysis, but whose effect was not mentioned in the paper. We did not include the missing values in the calculation of percentages. Average quality ratings between 1 and 1.4 were considered ‘strong’ (SQ), between 1.5 and 2.4, ‘moderate’ (MQ), and between 2.5 and 3, ‘weak’ (WQ).

The acculturation correlates were labeled by two of the authors in a consensus process. Variables that reflected high preference to preserve the ethno-cultural heritage were labeled as representative of the ‘maintenance’ acculturation strategy, whereas variables that reflected high preference to participate in the receiving community were labeled as ‘participation’ acculturation strategy. Acculturation is here defined as the complex, multidimensional, and individual process of psychological and social adjustment to a new cultural context [4, 35, 36].

#### Intersectionality

Due to the still incipient use of the intersectional approach in the study of Turkish and Moroccan immigrant populations, we used the single correlates as leading framework. Within the discussion of every single correlate, we mentioned the interplay with other correlates whenever this was examined by the study.

This study is part of a larger review study and reports henceforth only on the included papers that examine the prevalence and correlates of depressive disorders and depressive symptoms among the aforementioned groups. Other results of the described search are reported in another paper [13].

## Results

**Fig. 1:**
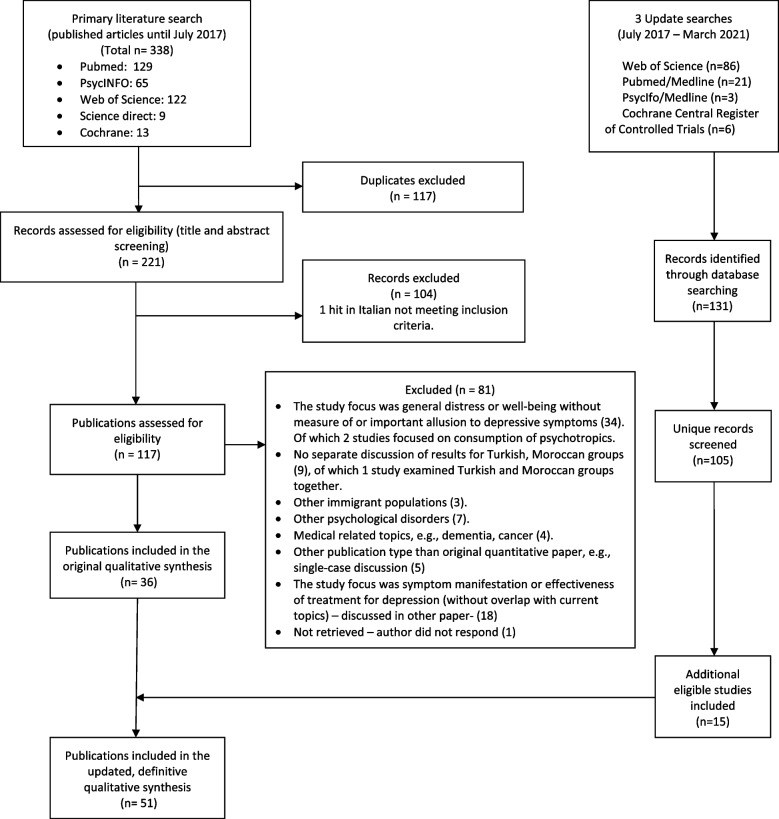
Flow diagram of the literature search and study selection

A total of 51 studies, published between 2000 and 2021, met inclusion criteria and concerned prevalence and correlates of depressive disorders and symptoms among Turkish and Moroccan populations in 6 (mostly Northwestern) European countries, namely Germany, The Netherlands, Belgium, Austria, United Kingdom and Spain. Our search did not retrieve relevant non-English or non-German records, and we excluded studies that included Turkish or Moroccan immigrants (of which 1 study examined both groups together) because their results were not discussed separately and thus were inconclusive for the purposes of this review (see Fig. 1).

All included studies used quantitative methods and a cross-sectional design regarding the prevalence and correlates. All studies included Turkish samples and the majority of these studies also included Moroccan samples. About forty percent of the studies with Turkish and sixty percent of the studies with Moroccan individuals examined community samples. The rest consisted of clinical samples or samples from the general population. Approximately half of the studies used solely a self-report instrument with a cut-off to determine a case. The majority of the included studies used a self-report instrument to measure depressive symptoms. About a third of the studies used both a self-report instrument and a semi-structured interview (i.e., CIDI, SCID-I, MINI), or only a semi-structured interview. Existing clinical records with a diagnosis were used in less than a fifth of the studies. Most studies conducted assessments in the participants’ preferred language by trained bilingual interviewers or using back-translated instruments. The majority of studies were of moderate quality (see Additional file 2 for the summary of the included studies and Additional file 4 for the detailed quality ratings). During the screening process of the update search, studies were excluded for several reasons, similar to those for the main search (Fig. 1). The most common were a lack of information on measured depressive symptoms and their related factors, no specific findings on Turkish or Moroccan groups and focus on other immigrant populations.

### Prevalence and comorbidity

In Table 1 we present the studies examining current one-month one-year and lifetime prevalence of depressive disorders, and one-year prevalence of comorbid depressive and anxiety disorders among Turkish or Moroccan immigrant populations in the Netherlands, Belgium, and Germany. The highest prevalence of depressive disorders was, in all three countries, found in the groups with Turkish backgrounds. Prevalence range was broad, spanning between 3.3% to over 68%, depending on the assessment criteria and sample characteristics (e.g. elderly subgroups). Those with Moroccan backgrounds had, if data were available, either elevated or equal depression levels compared to natives, with percentages ranging from 2.5% to about 43%.

**Table 1:** Overview of the prevalence rates organized by country and type of prevalence

						Turkish (%)			Moroccan (%)			Native-born (%)		
Country	Type of prevalence	Study	Instrument (cut-off) / Diagnoses	Population	Age (Range/ M ± SD)	Male	Female	All	Male	Female	All	Male	Female	All
Belgium	Current	Levecque et al., 2009^g^ [49]	SCL-90 (90% cut-off)	Community-based	18–65			15.4^d^			14.2^d^			9.1
Germany	Current	Morawa & Erim, 2014^c,g^ [50]	BDI (≥ 18 cut-off	Community-based, Ruhr region	40.0 ± 13.2			25.7^d^						7.9
		Sariaslan et al., 2014^c,g^ [51]	BDI (≥ 18 cut-off)	General practice patients	38.4 ± 12.3			19.3^d^						7.9
		Bermejo et al^h^., 2016 [52]	PHQ-D (≥ 11 cut-off)	Community-based	53.1 ± 11.0			30.4						
		Beutel et al., 2016^h^ [53]	PHQ-9 (≥ 10 cut-off)	General population, Western-mid region	35–74	14.8	35.1^i^	21.6^d^						6.8
		Fassbender & Leyendecker, 2018 [54]	CES-D-10 (≥ 10 cut-off)	Cross-sectional, community sample	35.9 ± 5.6		39.4 (lower income cluster), 43.3 (low education cluster), 28.1 (more advantaged cluster in terms of income & education)							
		Reich et al., 2018^h^ [55]	PHQ-9 (≥ 10 cut-off, moderate and severe symptoms)	Mixed general population, community and inpatient	42.6 ± 12.8			40.7						6.0
		Morawa et al., 2020 [56]	PHQ-9 (≥ 10 cut-off)	Community-based, General population	41.6 ± 11.3	26.4	33.2	29.2						
	One-year	Janssen-Kallenberg et al., 2017 [57]	CIDI DIA-X Version 2.8 (section E) MDD and/ or Dysthymia	Community-based Hamburg Berlin	18–65	23.2	33.2							
					18–29			22.3						
					30–49			27.3						
					50–65			40.6						
	Unclear	Erim et al., 2011 [58]	DSM-IV diagnosis MDD (code 296.2)	Psychosomatic outpatients secondary care	39.41 ± 9.9			37.3			-			-
			DSM-IV diagnosis Recurrent depression (code 296.3)					7.8						
			DSM-IV diagnosis All mood disorders (codes 296.2, 296.3, 300.4)					78.4						
The Netherlands	Current	Van der Wurff et al., 2004^g^ [37]	CES-D (≥ 16 cut off)	Community-based, non-institutionalized	55–64	47.2^d^	64.3^d,i^	55.5^d^	21.1^d,e^	45.2^d,e^ ^i^	31.9^d,e^	13.6	20.0	16.9
					65–74	64.4^d^	73.5^d^	68.8^d^	40.0^d,e^	46.3^d,e^	42.7^d,e^	2.6	20.2^i^	12.8
					55–74			61.5^d^			33.6^d,e^			14.5
		Ikram et al., 2015^b,h^ [38]	PHQ-9 (≥ 10 cut off)	Community-based Amsterdam	18–70			24.0^d^						5.8
			Algorithm PHQ-9	Community-based Amsterdam	18–70			12.9^d^						2.3
		Galenkamp et al., 2017^b,h^ [39]	PHQ-9 (≥ 10 cut off)	General population Amsterdam	18–70	18.8	26.4		18.2	22.0				
		Snijder et al., 2017^b^ [40]	PHQ-9 (≥ 10 cut off)	General population Amsterdam	18–70			23.0			21.0			
		Nieuwenhuijsen et al., 2015^b^ [41]	PHQ-9 (≥ 10 cut off)	General population, working subset Amsterdam	43.0 ± 12.0	13.0	21.0	16.0	10.0	17.0	13.0	3.0	6.0	5.0
		Stronks et al., 2020^b^ [42]	PHQ-9 (≥ 10 cut off)	General population Amsterdam	18–70			19.5 (offspring), 24.5 (immigrants)			15.5 (offspring), 22.0 (immigrants)			7.2
	One-month	De Wit et al., 2008^a^ [43]	CIDI 2.1 (section E)	General population Amsterdam	18–65			14.9^d^			6.6^d,f^			4.4
		Braam et al., 2010^a^ [44]	CIDI 2.1 (section E)	General population Amsterdam	19–82			18.0^d^			6.0^f,f^			5.0
		Schrier et al., 2010^a^ [45]	CIDI 2.1 (section E)	General population Amsterdam	47.3 ± 14.2; 49.6 ± 14.4; 54.1 ± 14.6			16.5^d^			5.8^d^			4.1
		Unlu et al., 2014^a^ [46]	CIDI 2.1 (section E)	General population Amsterdam	18–55			17.1						
			CIDI 2.1 (section E, D) Comorbid depressive and anxiety disorders	General population Amsterdam	18–55			6.7						
	One-year	De Wit et al., 2008^a^ [43]	CIDI 2.1 (section E)	General population Amsterdam	18–65			22.4^d^			9.8^d,e^			10.3
		Fassaert et al., 2010 [47]	ICPC diagnoses (codes P03, P76)	General practice patients	38.7 ± 11.3; 35.7 ± 9.3 53.2 ± 18.6			3.3^d^			2.5			2.5
		Schrier et al., 2012^a^ [45]	CIDI (section E) MDD and/or dysthymia	General population Amsterdam	47.5 ± 14.1; 49.5 ± 14.5; 54.1 ± 14.8			15.6^d^			4.3^d,e^			9.1
			CIDI (sections E, D) Comorbid depressive and anxiety disorders	General population Amsterdam	47.5 ± 14.1; 49.5 ± 14.5; 54.1 ± 14.8			9.8^d^			3.8^e^			2.3
		Fassaert et al., 2010 [17]	DSM-IV diagnosis (codes 296.21–296.24, 296.31- 296.34)	Outpatient and inpatient secondary care	35.4 ± 8.3; 35.3 ± 8.7; 40.6 ± 11.7			27.2			29.6^d^			25.5
	Lifetime	De Wit et al., 2008^a^ [43]	CIDI 2.1 (section E)	General population Amsterdam	18–65			31.1^d^			15.2^d,e^			24.8
	Point-prevalence	Fassaert et al., 2010 [17]	DSM-IV diagnosis Severe depression (codes 296.21–296.24, 296.31- 296.34)	Outpatient and inpatient secondary care	35.4 ± 8.3; 35.3 ± 8.7; 40.6 ± 11.7			32.1			34.2^d^			30.1
			DSM-IV diagnosis Recurrent depression (codes 296.21–296.24, 296.31- 296.34)	Outpatient and inpatient secondary care	35.4 ± 8.3; 35.3 ± 8.7; 40.6 ± 11.7			27.9^d^			25.4^d^			40.7

*Abbreviations*: *CIDI 2.1 section D*  anxiety disorders included social phobia, agoraphobia, panic disorders and generalized anxiety disorders, *CIDI 2.1 section E* depressive disorders included major depressive disorder and dysthymia, *ICPC*   International Classification of Primary Care, *MDD* Major depressive disorder, *M* Moroccan migrant group, *T* Turkish migrant group

^a^Studies based on the same sample/cohort

^b^Studies based on the same sample/cohort

^c^Studies based on the same sample/cohort

^d^Different from the native-born group

^e^Different from the Turkish group

^f^Non-specified relationship with other groups

^g^Current-prevalence (one week)

^h^Current prevalence (two weeks)

^i^Sex difference

This was the case in the Netherlands [17, 37–48], where the highest prevalence of depressive disorders was observed for Turkish-Dutch, followed by Moroccan-Dutch, while the lowest rates were observed in the native-Dutch group. These figures represented the estimates of mostly first-generation, low educated, Turkish and Moroccan individuals.

Taking also studies using self-report measures of depression (e.g. BDI, SCL-90, PHQ-9) into account, the one-month prevalence in the general population for Turkish-Dutch was significantly and consistently higher than the prevalence for native Dutch [37, 38, 42–45]. The one-month prevalence of depression among Turkish-Dutch was also higher than other immigrant groups’ prevalence [38] (including in some cases the Moroccan-Dutch [37, 43]. Furthermore, as shown in Table 1, the Turkish group had the highest lifetime (31.1%), one-year prevalence (22.4%) [43] and one-year prevalence of comorbid anxiety and depressive disorders (9.8%) in the general population [48] compared to Moroccan-Dutch and native Dutch. Turkish- Dutch also had the highest one-year prevalence (3.28%) in the general practice [47]. Moroccan-Dutch in the general population, especially older adults, had in most studies higher one-month prevalence rates [37, 43, 45] but lower one-year and lifetime prevalence than the native Dutch (9.8%, 15.2% vs. 10.3%, 24.8%) [43]. Only one study examined the Dutch secondary mental health care [17]. In this sample, Moroccan-Dutch patients showed a higher prevalence of severe depression than Turkish- or native Dutch. Moroccan- and Turkish-Dutch patients showed a higher prevalence of comorbid DSM-IV axis I disorders, but recurrent depression was more prevalent among native Dutch.

To summarize the Dutch data, taking the one-month prevalence rates as main indicators, we found that Turkish-Dutch of the general population have the highest prevalence of depressive disorders, which was higher that the prevalence of Moroccan-Dutch and native-Dutch. Moroccan-Dutch and native-Dutch did not differ in (rather low) prevalence rate. Practically the same patters were found regarding the one-year prevalence rates, but the lifetime prevalence showed that the Moroccan-Dutch had a much lower prevalence than both Turkish- and native Dutch (see Table 1). Nevertheless, it should also be noted that many of the current population studies have been largely based on the same samples.

The only Belgian study [49] was performed on a national community sample. The study showed that the prevalence of high-severity depressive disorder (90% cut-off on the SCL-90 depression subscale) was higher among both Turkish (15.4%) and Moroccan immigrants (14.2%) than among native Belgian (9.1%).

Nine German studies examined the prevalence of depressive disorders in Turkish-German immigrants [50–58]. The prevalence of clinically significant depressive disorders was higher than the prevalence of Polish-German individuals and norm values for the German population [50, 51, 53]. In separate gender analyses, Turkish-German women showed the highest prevalence of depression (35%) [53], which was even higher (about 40%) among economically and educationally disadvantaged women [54]. In samples of Turkish-German patients with psychosomatic symptoms, the prevalence of major depressive disorders was 37.3%, and there was a high comorbidity of mood disorders with somatoform disorders (43.9%-85.7%) and posttraumatic stress disorder (31.4%) [59]. Summarizing, the current prevalence of depressive symptoms for Turkish-German population was high and considerably higher than the prevalence of the native-German (based on norm values).

### Correlates of depressive disorders and symptoms

The results of the box-analysis in Table 2 show the associations included in studies examining the relationship between psychological characteristics, socio-demographic factors, ethnocultural aspects, and depressive symptoms.

**Table 2:** Box analysis of variables examined in relation to depressive symptoms

			Association (+ , -, 0, ?) / Average quality per association type		
Category	Factor	Subscale	Turkish	Moroccan	Native-born
Psychological characteristics	Personality	Neuroticism	+ / 2	+ / 2	+ / 2
		Extraversion	- / 2	- / 2	- / 2
		Openness	- / 2	- / 2	- / 2
		Consciousness	0 / 2	0 / 2	0 / 2
		Agreeableness	- / 2	- / 2	- / 2
	Emotion regulation	Emotional intelligence	- / 3^a^	- / 3^a^	
		Emotional coping (expression and emotional processing)	+ / 3		
		Positive coping (hope, optimism)		-, - / 3	-, - / 3
		Cognitive reappraissal	- / 3 0 / 3		- / 3 0 / 3
		Expressive suppression	+ /3 - / 3		+ , + / 3
	Self-appraisal	Positive—Self-esteem, self-efficacy	- / 3	- / 3	-, - / 3
		Negative—loss of status, self-deprecation (guilt), shame	+ ^e^, + ^e^, + ^e^ / 3		+ ^e^ /3 0^e^, 0^e^ /3
	Sense of control	sense of coherence^f^ and sense of mastery	-, -/ 1.5	- /1	
	Attachment/ Autonomy	Independent self	- / 3		- / 3
		Autonomy-satisfaction	- / 3		+ / 3
		Desire of independency	0 / 3		0 / 3
	Attachment/ Relatedness	Interdependent self	- / 3		- / 3
		Relatedness-satisfaction	- / 3		0 / 3
		Separation anxiety	0 / 3		+ / 3
		Fear for intimacy	+ / 3		+ / 3
		Trusting problems	+ / 3		+ / 3
Socio-demographic factors	Sex	Female	+ , + , + , + , + , + , + , + , + ^e^, + , + / 1.9 0, 0, 0, 0, 0, 0, 0, 0^a^, 0 / 2.3 ?, ?, ?, ?, ?	+ , + , + , + / 1.8 0, 0, 0, 0 / 2.3 ?	+ , + , + , + ^e^ /1.8 0, 0, 0, 0, 0, 0, 0, 0 / 2.1
		Not specified	0 / 1	0 / 1	
	Age		+ , + , + , + / 1.8 0, 0, 0, 0, 0, 0, 0, 0, 0, 0, 0, 0, 0, 0^a^, 0, 0, 0 / 1.9 ?, ?, ?, ?, ?	+ /1 0, 0, 0, 0, 0, 0, 0, 0 / 1.9 ?	+ , + /1 0, 0, 0, 0, 0, 0, 0 / 1.9 ?^e^
	SES	Combined variable	-, - / 3		
		Education	-, -, -, -, -e, -, -, - / 2 0, 0, 0, 0, 0, 0, 0, 0, 0 / 2.3 ?, ?, ?	-, - / 1.5 0, 0, 0, 0, / 1.5	0, 0, 0, 0, 0, 0, 0, 0, 0/ 2 ?^e^
		Not specified education	+ / 1		
		Unemployment	+ , + , + ^e^, + , + / 2.2 0, 0, 0, 0/ 2.3 ?	+ /2 0, 0 / 1.5	0 /2
		Not specified employment status	+ / 1 0^a^ / 2		0 / 1
		Occupational level (categories low to high)	- / 2	- / 2	
		Income (unidimensional)	- / 2 0, 0, 0, 0, 0 / 1.4	0, 0, 0, 0 /1.3	0, 0 / 1.5
		Negative financial indicator (financial problems, poor housing, no home ownership, retired, daily hassles)	+ , + , + / 2.7 0, 0 / 1.5	0 / 2	
	Marital status	Not specified	+ / 1 0, 0, 0 / 2.3 ? / 2	0 / 2	0, 0 / 2
		Single/living alone	+ , + , + / 1.3 0, 0, 0, 0^a^/ 2	+ / 1 0 / 1	+ / 1 0 / 3
		Marriage duration	0 / 3		
		Poor marriage quality, marriage problems	+ , + , + / 2.3 0, 0 / 3		
	Role functioning/disability	Physical limitations	+ / 1	+ / 1	+ / 1
		Chronic illness	+ / 1	+ / 1	+ / 1
		Physical activity	? / 2		
		Daily functional performance	0^a^ / 2 + / 2	+ ^a^, + / 2	0^a^ /2 + / 2
		Trauma/PTSS	+ , + ^a^ / 2.5		
		Perceived stress	+ ^c^ / 2		
		GAF	-^e^ / 3		?^e^ / 3
		Clinical treatment duration	+ ^e^ / 3		?^e^ / 3
		Attitude towards treatment (psychotherapy, medication)	0 / 2 + / 2		
		Quality of life, satisfaction with life	-, - / 2.5		-/ 2
Ethnocultural factors	Ethnicity		+ , + , + , + , + , + , + , + , + / 2 0,0, 0 / 2.3	+ , + , + , + , + , + / 1.7 0,0, 0 / 2	0, 0, 0, 0, 0, 0 / 2.2
	Religiousness	Positive religiosity all types	0^a^, 0, 0, 0 / 1.5	-, - / 1 0 / 1	
		Positive coping	0 / 1	0 / 1	
		Practicing religion	0^a^ / 3		
		Daily prayer	0 / 1	- / 1	
		Service attendance	0 / 1	- / 1	
		Negative coping all types	+ , + / 1 0, 0, 0 / 1	+ , + / 1 0, 0, 0 / 1	
		Negative – coping without God	0 / 3	0 / 3	
		Negative – doubt about God	0 / 3	0 / 3	
		Negative – anger at God	+ / 3	0 / 3	
		Believing being punished by God	0 / 3	+ / 3	
		Believing being abandoned by God	+ / 3	+ / 3	
	Acculturation	Unidimensional measure	-^a, c^ / 3 0^a^ / 3		
		Participation (incl. assimilation, integration, skills, social integration, satisfaction in the receiving country, citizenship, assimilated social network)	+ ^a^. + / 2.5 -^a^, -^a^, -, -, - / 2.4 0, 0 / 2	0^a^, 0^a^ / 2 + / 2	
		Maintenance (incl. separation, marginalization, traditions, norms and values, feelings of loss, homesickness, same- ethnic contact, number of same-ethnic friends, same-ethnic visits, same-ethnic shopping, speaking mother tongue with friends, communication problems, frequency of visits to Turkey/Morocco, only negative reasons to stay in receiving country, marginalized social network)	+^a^, +^a^, +, +, +, +, +, +, +, +^e^, +, +, + / 1.9 -, - / 1 0^a^, 0^a^, 0^a^, 0^a^, 0, 0 / 2.2	0^a^, 0^a^, 0^a^, 0, 0, 0, 0, 0, 0 / 1.3 + , + , + / 1.7	+ ^e^ / 3 0^e^ /3
		Language proficiency (language of receiving country)	- / 2 0, 0, 0, 0, 0, 0 / 1.7		
		Age of migration	0,0 / 1.5		
		Stay duration	0, 0, 0, 0, 0 / 2.2		
		Ethnic identity	-^b^, - , - / 20^a^ / 1	- / 2 0 / 1	
	Problematic migration		+ , + ^e^ / 2	0 / 1	0^e^ /3
	Migrant status		0 /2		
	Generation	First	+ / 2 0, 0 / 1.5	0 /2	
		Second	+ / 3	+ / 3	
		Not specified	0 / 1 ?, ?	0 / 1	
	Social support	All types	-, -, -, -/ 1.5 0, 0, 0, 0/ 1.8	-, - / 2.5 + / 1 0, 0 / 1	- / 3
		Available	- / 3		
		Perception of social support	- / 1	- / 2	
		From friends and neighbors	0 / 2		
		From family members	0 / 2		
		In the spare time	0 / 2		
		Same-ethnic contact^d^	-, - / 1 0 / 1	+ / 1 0, 0 / 1	
		Assimilated social network (contact with natives)^d^		+ / 2	
		Problems with or within social network (psychological, medical treatment, departure, incarceration of family member, family problems)	+ , + , + / 2 0 / 2 + ^e^ / 3		+ ^e^ / 3
		Forced marriage (and related problems)	+^a^, + ^e^ / 3		0^e^ /3
		Loneliness, marginalized social network^d^	+ , + ^d^ /1.5	+ , + ^d^ /1.5	+ /1
	Perceived ethnic discrimination	All types	+ , + ^a^, + , + , + , + , + , + ^e^, + , + / 1.7 0, 0 / 1	+ , + , + , + / 1.3 0 / 1	0^e^ /3
		Not specified discrimination	+ , + . + / 1.3	+ , + / 1.5	
		Personal-level	+ / 1	+ / 1	
		Group-level	0 / 1	+ / 1	
		Open aggression	+ / 2		
		Passive /subtle discrimination (i.e., paternalism)	+ / 2		
		Everyday discrimination (neighborhood, doing groceries, at work, in public institutions)	+ / 2 0 / 1		
	Work environment	Recovery opportunities	- / 3	- / 3	0 / 3
		Perceived stress	0 / 3	- / 3	0 / 3

^a^Bivariate analysis

^b^Moderating effect

^c^Mediating effect

^d^Variable also considered as part of acculturation variables

^e^Differences in migrated-related stressors between ethnic groups in a population with depressive symptoms

^f^sense of coherence understood as the appraisal of the world as comprehensible, manageable and meaningful

Abbreviations: 0 = non-significant association, —= negative association with depressive symptoms (lower level of depressive symptoms), + = positive association with depressive symptoms (higher level of depressive symptoms), ? = not specified (missings), 1–1.4 = strong quality (SQ), 1.5–2.4 = moderate quality (MQ), 2.5–3 = weak quality (WQ), SES = Socioeconomic status, GAF = Global Assessment Functioning

### Psychological characteristics

#### Personality

One MQ study found that Turkish- and Moroccan- Dutch (mostly first generation) community individuals showed the same personality profile (high neuroticism, low extraversion, agreeableness, and consciousness) related to higher depressive disorders seen in native Dutch [60]. The association with depressive symptoms was stronger for neuroticism for Turkish-Dutch compared to Moroccan- and native Dutch, whereas the same was the case for lower agreeableness of Moroccan-Dutch compared to Turkish- and native Dutch, also after controlling for gender, age, education and employment [60].

#### Emotion regulation

Three (WQ) German studies with mostly first-generation, Turkish-Germans examined emotion regulation in community and clinical samples [61–63]. Findings showed that in healthy controls and community samples, emotional suppression [61] and higher emotional intelligence (ability to recognize, regulate and observe own's and other people’s emotions; [62]) were related to lower levels of depressive symptoms. Whereas emotional coping (i.e., emotional expression and processing) was related to higher levels of depression for Turkish-German [63]. The study examining the clinical sample showed that in this population cognitive reappraisal was protective, whereas emotional suppression was related to higher levels of depression [61]. Summing up, emotional suppression seems protective for community samples, whereas a balanced use of both cognitive reappraisal and emotional expression seems protective for clinical Turkish-German samples.

#### Self-appraisal

Two (WQ) German studies inform us on the relationship between positive and negative aspects of self-appraisal and depressive symptoms. They found that positive self-esteem was protective among a Turkish-German community sample with 20% second-generation participants [63], whereas negative aspects of self-appraisal, such as guilt and shame were significantly more pronounced among Turkish-German inpatients of first- and second-generation with depressive symptoms than among native-German inpatients [64]. Guilt was especially prevalent among Turkish-German women. Loss of status was highly reported, especially by male inpatients of both immigrant and native German background [64].

#### Sense of control

Results of two (average MQ) studies examining a medium-sized Turkish-German clinical sample and a large representative Turkish- and Moroccan- Dutch sample of the general population showed that having a high sense of individual mastery [65] and of coherence (i.e., experiencing the world as comprehensible, manageable and meaningful; [58]) predicted lower levels of depressive symptoms. Sense of mastery mediated and moderated the relationship between perceived ethnic discrimination and depression (see the subsection ‘discrimination’ for further discussion; [65]). Higher levels of self-efficacy, hope and optimism were associated with lower depression scores in a WQ study with a sample of young men with a Moroccan background in Spain [66].

#### Attachment

Three WQ studies examined autonomy and relatedness aspects of attachment [67–69]. Two German studies showed that a balance between both satisfaction with autonomy as with relatedness [67], and identification with both independent and interdependent self-construal (i.e., the definition of the self is both formed by individual and relational experiences; [68]) predicted lower depressive symptomatology in first-generation, female, Turkish-German, clinical populations, also after controlling for socio-demographic variables. One of these studies found that whereas satisfaction with autonomy is most relevant for healthy native-Germans, and satisfaction with relatedness is most relevant for healthy Turkish-Germans, the most important difference between Turkish-German patients and immigrant healthy controls was the higher satisfaction with autonomy reported by the healthy controls [67]. In opposition, one Austrian study [69] showed that even though Turkish-Austrian patients showed lower separation anxiety and desire of independency, and higher trusting problems and fear of intimacy than native- Austrian patients, the Turkish-Austrian patients did not differ from the Turkish-Austrian healthy controls [69]. Summarizing, there is evidence that immigrant populations differ from native populations in attachment style; however, the evidence showing that aspects of attachment discriminate immigrant patients from healthy controls is mixed.

### Socio-demographic factors

#### Sex

The results regarding female sex were mixed for Turkish and Moroccan groups (see Table 2). A slight majority of the studies examining sex in Turkish samples [37, 38, 43, 49–51, 53, 57, 64] found female sex positively related to depressive symptoms in German, Belgian and Dutch community and patient samples. A community-based German study [57] showed that women with German or Turkish-German ethnic identification (vs. Turkish identity) or a low education level were more likely to report depressive symptoms. Among Dutch- and Belgian Moroccan mainly community samples, about half of the studies found female sex positively related to depressive symptoms [37, 41, 49, 65]. On the other hand, also about the half of the studies examining large Moroccan-Dutch and Turkish community and patient populations of Germany, The Netherlands, and the UK found sex neither to be related to nor predictive of depressive symptoms [43, 52, 56, 59, 60, 63, 70–72]. Summing up, the findings regarding sex are rather inconclusive, showing a small tendency towards female sex being more often related to depressive symptoms in Turkish groups with lower education.

#### Age

The vast majority of the studies examining age in Turkish or Moroccan groups found no relationship with depressive symptoms in Belgium [49], the Netherlands [43, 60, 71], Germany [50, 56, 59, 63, 72–74] and UK [75]. The remaining studies showed mixed results, stating older age as a predictor of higher depressive symptomatology in Turkish-German [51, 53, 57] -Dutch populations [37].

#### SES

Despite the fact that in most studies Turkish and Moroccan individuals, compared with native and with other immigrant groups, had a lower SES (as indicated by education level, employment status, income, and/or homeownership [37, 38, 44, 49, 51, 60, 61, 67], most of the examined associations in the studies with Turkish populations found that SES was not related to, nor predicted depressive symptoms. Especially education level and income in Turkish-German, -Dutch and -Belgian samples were often not related to depression [37, 43, 44, 49, 50, 59–61, 63, 67, 68]. Unemployment was not related to depressive symptoms in about 50% of cases [49, 60, 72–74]. On the contrary, the study of Stronks et al. [42] in the Netherlands, showed that the prevalence of depression was substantially higher for people with immigrant status (both Turkish and Moroccan) without paid jobs, especially for men and people who were incapacitated or had no access to the labour market, indicating that the association between depressed mood and SES-factors was not linear. About a quarter of the examined associations found that SES appeared related to higher depressive symptomatology in mostly Turkish clinical samples [50, 53, 59, 74, 76]. Summarizing the results for Turkish populations, only about a fifth of the investigated associations showed that SES factors were related to higher levels of depression (see Table 2).

Most of the studies on Moroccan-Belgian and -Dutch community samples found no relationship of depressive symptoms with education level [43, 44, 49, 60], employment status [49, 60], income, or home ownership [37, 43, 44, 49].

#### Marital status

Most examined associations showed that marital and household status were not related to depressive symptoms in Turkish and Moroccan samples [46, 49, 59, 67, 68, 72]. In three SQ studies, being single or living alone showed a detrimental effect in an older, community Moroccan-and Turkish- Dutch sample [37], in community and clinical Turkish-German samples [50], and in Turkish-German women of the general population [56].

#### Role performance/disability

One SQ study showed that, in older adults from community samples, physical limitations and chronic illness predicted a higher level of depression [37]. This finding was not related to ethnicity, but the effect of physical limitations was less strong for the Turkish groups [37]. Depression severity was related to lower daily functioning only among older Moroccan-Dutch adults in one MQ study [77] and predicted functional disability in all ethnic groups in other MQ study (no ethnicity interaction effect), also after controlling for age, sex and education [45].

In four (average MQ) studies, traumatic experiences [78, 79], and perceived stress [73] were related to higher severity of depression in Turkish-German general practice patients and in patients with psychosomatic complaints. Depressive symptoms were also negatively associated with quality of life of Turkish-German patients with depression [80].

One (MQ) study [81] reported the attitude towards medication and psychotherapy of Turkish-German outpatients, who reported a significantly more positive attitude towards medication than patients without migration background. Higher scores of depressive symptoms were associated with positive attitude towards medication. When controlling for sociodemographic and clinical variables, no significant differences in attitude towards psychotherapy were reported. Acculturation neither influenced the attitude towards psychotherapy nor towards medication.

### Ethnocultural factors

#### Ethnicity

Besides the earlier described prevalence studies, the majority of studies with Turkish and Moroccan samples that explicitly included the variable ethnicity in their multivariate models, showed that ethnicity was a salient predictor of depressive symptoms, depressive disorders or risk of depression treatment. Ethnicity appeared an equally or more important factor than other correlates, such as gender, age or SES [37, 43, 51, 53, 71]. This was the case in the German and Dutch general population [43, 71], and clinical [71], and general practice samples [51]. In Belgium, ethnicity was not related to depressive symptoms in a study excluding natives [49].

#### Religion

Three (average MQ) studies found that positive religious behaviors and coping were not associated with depressive symptoms in Turkish-Dutch and –German community and general samples [44, 63, 81]. Two SQ studies showed that Moroccan-Dutch practiced religion the most, which was strongly related to a lower level of depression [44, 82]. Some types of negative religious coping were predictors of higher levels of depressive symptoms for either one or both ethnic groups: feeling anger towards God (Turkish), believing being punished by God (Moroccan), and believing being abandoned by God (both) [44] (see Table 2).

#### Social support

In the Turkish-Dutch population, 50% of the MQ studies, showed that time spent with and the number of same-ethnic friends predicted indirectly lower levels of depressive symptoms by weakening the association between perceived ethnic discrimination and depression [82]. Also, problems of members within the social network predicted higher depressive symptomatology [76]. The results for Moroccan populations were scarce and inconclusive. General social support predicted lower depressive symptomatology in a Moroccan-Belgian sample [49], and it was associated to lower depressive symptoms among young, Moroccan-Spanish men [66]. In Dutch samples, both having more contact with same-ethnic people than with natives [37], as having more contact with natives than with same-ethnic people [42] were associated to more depressive symptoms. The former was especially important for older individuals.

#### Acculturation

Acculturation is the complex, multidimensional, and individual process of psychological and social adjustment to a new cultural context [4, 35, 36]. Berry’s [83] bidimensional model of acculturation is one of the most influential frameworks delineating two separate dimensions: maintenance of the culture of origin (maintenance) and adaptation to the receiving culture (participation). The combination of these dimensions results in four acculturation strategies: integration (high maintenance—high participation,), assimilation (low maintenance—high participation), separation (high maintenance—low participation), and marginalization (low maintenance—low participation). Most of the examined studies used unidimensional (one spectrum ranging from unacculturated to acculturated) or multidimensional (single, separate dimensions of acculturation e.g. traditions, feelings of loss) acculturation models [84], or proxy measures of acculturation (e.g. receiving country language proficiency, stay duration). We related the multidimensional variables to the main ‘maintenance’ and ‘participation’ dimensions, according to Berry’s [83] bidimensional model (e.g., ‘homesickness’ as a concept was considered to be a sign of ‘maintenance’, whereas ‘satisfaction in the receiving country’ was considered a sign of ‘participation’).

##### Acculturation strategies 

Individuals who endorsed maintenance, separation or marginalization acculturation strategies were more often of the first generation, and showed stronger (Turkish) ethnic identity, better (Turkish) ethnic language proficiency, lower proficiency in the language of the receiving country, lower education level, lower income, and older age [42, 50, 85]. Contrarily, longer lasting intimate relationships, younger age, higher education level [46], second generation [40, 52], and higher emotional intelligence [62] were associated to participation, integration or assimilation acculturation strategies.

We considered the acculturation related results explicitly regarding ethnic and sex differences:

##### Sex

Female sex was associated to lower participation in a clinical German sample [50], but this association was non-significant in a Dutch, employed, community sample [46]. Morawa et al. [50, 56] showed that separation as acculturation style among migrants of Turkish background in Germany was associated with higher levels of depressive symptoms in both genders.

##### Ethnicity

Compared to Moroccan- and Surinamese-Dutch outpatients, the Turkish-Dutch outpatients showed the least well-acculturated position, both with respect to the ‘participation’ dimension (least skills, lowest social integration) as to the ‘maintenance’ dimension (most maintenance of cultural norms, values, traditions, and feelings of loss) [86]. Most of the MQ associations examining aspects of the maintenance acculturation strategy were related to, or predicted, higher risk or levels of depressive symptoms in Turkish-Dutch, as well as Turkish-German community and clinical samples [42, 46, 50, 56, 63, 64, 86] also after correcting for SES [46, 50]. Particularly, a marginalized style in terms of cultural orientation, social network and ethnic identity was related to a higher risk of depressed mood [42]. Only maintenance related social support aspects, like spending leisure time with same-ethnic people, predicted lower depression severity [82]. About a third of the associations concerning proxy variables of the maintenance acculturation strategy (e.g., use of mother tongue, contact with Turkish friends) were not related to depressive symptomatology [37, 57, 75, 86]. Regarding unidimensional acculturation and aspects of the ‘participation’ acculturation strategy, over half of the examined associations in Turkish samples showed that higher acculturation and participation variables, such as social integration and satisfaction in the receiving country, predicted lower risk, or lower level, of depressive symptomatology [46, 50, 52, 57, 62, 86]. Other acculturation related variables, such as age of migration [50, 74], duration of stay in the receiving country [50–53, 57, 59, 74] and proficiency in the language of the receiving country [51, 52, 56, 57, 74, 76], did not predict depressive symptoms in Turkish community or general practice samples.

Moroccan-Dutch outpatients showed a medium–high degree of both participation and maintenance acculturation and reported an intermediate acculturation level compared to Turkish- and Surinamese-Dutch [86]. The vast majority of the examined SQ associations showed that neither maintenance nor participation acculturation variables nor ethnic identity of Moroccan-Dutch patients were associated to, or predicted, depressive symptoms [37, 81, 86].

##### Summarizing

In Turkish samples, more than half of the associations regarding aspects of the maintenance acculturation strategy showed that they were related to higher levels of depressive symptoms, whereas most of the associations concerning variables of participation acculturation showed that these variables were related to lower levels of depressive symptomatology. In Moroccan populations, acculturation variables were in most cases non-related to depression levels.

#### Generation

Most studies showed inconclusive trends though two MQ studies found no association between first generation and depressive symptoms in a Turkish and Moroccan community sample [49, 56].

#### Discrimination

Perceived ethnic discrimination has been defined as the perceived unfair treatment related to ethnic background [87]. Most studies including Turkish and Moroccan groups showed a strong association between different types of (perceived) discrimination and (higher) depressive symptomatology, also after controlling for socio-demographic variables [38, 42, 70, 73, 74, 81]. Perceived personal-level discrimination predicted up to three times higher depression prevalence in young, mostly second-generation, mid-highly educated, Turkish-, and Moroccan-Dutch individuals of community samples; this association seemed to be stronger for Moroccan-Dutch, for whom perceived group-level discrimination at school also predicted depressive symptoms [70]. Only one MQ study with a community sample of Turkish-German people found that perceived discrimination was not related to depression severity, despite the higher report of discrimination experiences [74].

Religion weakened the association between perceived discrimination and depressive symptoms of Moroccan-Dutch [82]. A strong ethnic identity of Turkish- and Moroccan-Dutch immigrants did not affect the relationship between daily perceived discrimination (overt and subtle) and depressive symptoms [82], but it canceled the indirect association between paternalism and depressive symptoms for mid-highly educated, Turkish-German with high Turkish identification (especially ethnic pride) [73].

## Discussion

This systematic review aimed to synthesize and evaluate the existing empirical literature on the prevalence and correlates of depressive disorders and symptoms in two of the largest immigrant groups in Northwestern Europe, namely the Turkish and Moroccan communities. Doing so, we used whenever possible an intersectionality approach by highlighting the more vulnerable as well as the most resilient subgroups within these populations related to the interaction of socio-demographic, psychological and/ or ethnocultural factors.

Regarding the prevalence, it is difficult to draw robust conclusions, especially for the Moroccan population. Even though the average quality of the epidemiological studies was moderate, the external validity of the findings was suboptimal due to low response rates, exclusive urban settings, and immigrant samples mainly comprising first-generation respondents. Taking these limitations into consideration, the results provided evidence that Turkish immigrants (with the strongest evidence for Turkish-Dutch, and -German older adults, women, and outpatients with psychosomatic complaints) consistently showed the highest (three-fourfold) prevalence of current and one-month depressive disorder compared to natives and, in some studies, to Moroccan and other immigrant groups. Their one-year and lifetime prevalence were also higher than those of native and Moroccan-Dutch.

The general picture that arose from the Dutch data regarding one-month and one-year prevalence of depressive disorders in Moroccan-Dutch is that they showed similar (rather low) prevalence rates to native Dutch. Moroccan-Dutch also had lower lifetime prevalence than the natives. Some exceptions were found for specific subgroups such as patients with severe depression and older adults. Also, Moroccan- Belgian had higher prevalence of depressive symptoms than their native counterparts.

The findings that Turkish immigrants had overall elevated prevalence rates of depression and that Moroccan immigrants, including young men, had rather moderately (or not elevated at all) prevalence rates were remarkable. A recent international study showed that the lifetime-prevalence of major depressive episode was 14.1% in Belgium, 9.9% in Germany and 17.9% in The Netherlands (based on communiy samples; [1]). Contrasting with these figures, though not all too well comparable, the one-month prevalence of Turkish immigrants (mainly patients) was generally well above the German prevalence. In the Netherlands the prevalence of Turkish immigrants laid above, and the prevalence of Moroccan immigrants laid under the reported prevalence of 17.9%. In Belgium, the current prevalence of depressive symptoms in both Turkish and Moroccan immigrants was about the same as the lifetime prevalence found in the international study; however, this may indicate that the lifetime prevalence of the Belgian immigrant population is higher.

Comparing the prevalence rates presented in this review with the rates in Turkey and Morocco, Turkish immigrants showed a higher one-year prevalence than Turkish in Turkey (8.2%; [88]). The opposite was found for the Moroccan group. Moroccan immigrants also reported lower rates of depression than Moroccans in Morocco, who showed a point prevalence of depressive disorders of 26.5% in a community sample [89] and 13.7% in the general practice [90]. There are authors that sustain that the level of depression of immigrant populations is not only related to adversity during migration, but also to adversity in the place of origin, before the migration (e.g., [6]). The finding that Turkish immigrants showed higher levels of depression compared to Moroccan immigrants might be comprehensible considering that in the more recent decades, Turkey has faced more political instability (including persecution of some groups) than Morocco [91, 92], which might have affected the mental health of the immigrants- to be. Other explanation might be that the symptoms of Moroccan immigrants are currently encapsulated in other disorders because (cultural) diagnostic problems. Recent studies showed that there is a tendency to under-detect mood symptoms and over-detect positive psychotic symptoms in Moroccan-Dutch at risk for psychosis [93, 94], and that using a culture-sensitive assessment instrument can help correcting the diagnostic bias in Moroccan-Dutch [95].

The prevalence in the Dutch general practice (based on GP’s assessment) was much lower compared to the prevalence in general or clinical samples and also compared to the German general practice (based on BDI’s cut-off). This finding is consistent with literature discussing underdiagnosis of moderate and severe cases of depression in the general practice, especially based on clinical interviews [96]. Contextual factors (differences between the Dutch and German mental health care system) might also be explanatory.

It is possible that the prevalence results are related to the methodological heterogeneity of the studies. For instance, there is evidence that the CIDI has construct, method and item bias in the older adults of the target groups [97]. Among others, the CIDI does not assess non-Western manifestations of depression [13]. The cross-cultural properties of the other used instruments (e.g. BDI, HADS) for Turkish-and Moroccan immigrants are unknown, excepting for the CES-D and PHQ-9. The CES-D shows good convergent validity and reliability, but another factor structure than the original as well as some item bias [98]; whereas the PHQ-9 shows measurement invariance between ethnic groups [39]. Currently, there is only some mixed evidence that despite the higher depressive symptomatology, Turkish, and Moroccan-Dutch report the same functional impairments as natives [45, 77]. Normative cut-offs of instruments to establish caseness, severity and functional impairment are still lacking. All these persisting methodological shortcomings plead for more research and assessment development on these aspects.

Research on correlates of depression for Turkish and Moroccan immigrant groups was available, but many factors were only examined once, which greatly affected the reliability of the conclusions. The richest and strongest evidence was found for socio-demographic factors and also some ethnocultural aspects, such as ethnicity, acculturation strategy, and perceived ethnic discrimination. It is worth noting that individual psychological correlates were scarcely examined, mostly in Turkish samples, and in rather low-quality studies. For Turkish community individuals, we found some indications for positive associations of high neuroticism (strong effect), and low extraversion, consciousness, and agreeableness with depressive symptomatology. On the other hand, social support, satisfaction with relatedness, self-esteem, emotional intelligence, and flexible use of emotion regulation strategies (both expressive suppression and cognitive reappraisal) related negatively to depressive symptoms, especially for Turkish-German women. In clinical Turkish samples an interdependent self-construal and sense of coherence were negatively related to depressive symptomatology. For Moroccan groups, high neuroticism, low extraversion, low consciousness, low agreeableness (strong effect), self-efficacy and both same-ethnic contact and more contact with natives predicted depressive symptoms. Other types of social support predicted lower levels of depressive symptoms.

Regarding socio-demographic and ethnocultural factors, ethnicity was relatively one of the most consistent factors associated with or predictive of depression in Turkish and Moroccan communities (also taking the prevalence studies into account). Female sex and being single or living alone appeared sometimes (about 40–50% of the times), positively related to depressive symptoms in Turkish and Moroccan samples. SES, age, and migration generation appeared least frequently related to depressive symptoms in both groups. For two reasons this was unexpected. First, most included studies showed a higher report of SES strains in Turkish and Moroccan than in natives or other immigrant groups. Second, international evidence points to strong associations of female sex, low SES position, older age, and first generation with increased rates of depression (e.g., [1, 6, 20]). Possible explanations for the unexpected findings could lie in quality and methodological artifacts. In this review, SQ studies with larger and more representative samples, and studies examining models with only socio-demographic correlates more often found that female sex and SES variables were predictors of depressive symptoms. In addition, the small variability of SES factors (floor effect) may have hampered some analyses. Also, studies examining models including ethnocultural correlates and other variables such as personality or sense of coherence showed that socio-demographic factors were unrelated to, or only had a limited direct relationship with depressive symptomatology. Socio-demographic factors seemed to play a stronger mediating or moderating role related to, or leading to depressive symptomatology, through other factors such as exclusion, isolation or adaptation problems [6]. However, the evidence on the moderating and mediating role of socio-demographic factors is too scarce to draw any strong conclusion.

An important finding that emerged was that perceived discrimination was a common experience of Turkish- and Moroccan-Dutch, especially young adults. Ethnicity predicted perceived discrimination (overt or subtle), which subsequently appeared as an utterly salient, strongly predictive factor of depressive symptomatology. The relationship was direct and indirect through lower religiousness (for Moroccan) and perceived stress, which according to the authors may arise due to incompatibility between internal (being German) and external self-perception (Turkish immigrant), especially among those reporting low Turkish ethnic identity [73]. This finding is congruent with the extended literature associating chronic stress due to experiences of discrimination and other factors common to immigrant populations, such as low SES, social isolation, and integration problems, with poor mental and physical health (e.g., [87, 99]). Studies have shown that biological dysregulation of the stress systems due to sustained exposure to stressors might be partly responsible for the negative effects on health (see for more information [100–102]).

In this review, evidence showed that the acculturation process and their associations with depressive symptoms differed per immigrant group. In line with acculturation research [103], low levels of maintenance acculturation strategies and high levels of participation strategies were most related to lower depressive severity among Turkish community samples. The notion that the combination of both lower maintenance and high participation acculturation strategies might be the most protective acculturation strategies for Turkish immigrant population is important, given that there are reports that this group currently tends to adopt maintenance strategies [86]. Our finding that duration of stay and receiving-country language proficiency did not predict depressive symptoms might be due to studies excluding respondents who did not master the receiving-country language (e.g. [53, 86]). The acculturation style of Moroccan-Dutch patients appeared unrelated to depressive symptoms, possibly because of their already mild acculturation style, less oriented to their own group and more to the receiving society (compared to the Turkish-Dutch style; [86]).

### Implications for clinical practice

#### Prevalence and diagnostics

Compared to natives, specific Turkish subpopulations that had a higher prevalence of depressive disorders and symptoms were women, older adults, and clinical populations; whereas vulnerable Moroccan subpopulations were older adults and patients with severe depression [37, 43, 45]. Clinicians are advised to ask about symptoms of depression in these groups in particular. The lower prevalence rates in the Dutch general practice [47] commends attention for the diagnostics of depressive disorders, especially among men, in this setting. We also recommend clinicians to pay attention to also highly prevalent comorbid anxiety [48], other depressive disorders, and somatoform and posttraumatic stress disorder [17, 58], which have been related to more severe and persistent psychopathology [48] and to negative treatment outcomes [86, 104]. Using available assessment instruments such as the DSM-5 Cultural Formulation Interview (APA; [105]) besides standardized diagnostic instruments to assess depressive symptoms might be advisable to improve recognition of symptoms and other relevant factors.

#### Psychological factors

Based on the results on correlates related to lower levels of depressive symptoms among Turkish populations, interventions aimed at reinforcing a combined interdependent and independent self-construal [68] as well as both satisfaction with autonomy and relatedness [67] could be pursued in therapy. Additionally, the treatment for depression for Turkish immigrant populations might be enriched by training to learn balancing cognitive reappraisal and expressive suppression as emotional strategies [61]. Interventions seeking self-empowerment (including a stronger sense of coherence, hope and self-efficacy) could be introduced, always in careful agreement with the patients, their environment, and the expectations of other parties involved. It also seems advisable to pay attention to signs of homesickness [63], feelings of loss [86], and/or traumatic experiences [78, 79], especially among older, Turkish, labor immigrants and their spouses.

#### Socio-demographic factors

Even though this effect was present on its own, studies showed that both groups also faced many social strains (e.g., unemployment, lower education, lower income; [37, 38, 42, 44, 49, 51, 60, 61, 67]). Exploration of the patients’ SES situation and how it relates to their daily life and experience of depression seems of added value in clinical practice in order to select possible targets of therapeutic interventions. Additionally, clinicians might do well to keep in mind that some SES factors might be more relevant for Turkish women (e.g., financial problems, being single) or for Turkish men (e.g., being unemployed; [76]). As (marital) relationships appeared to be somewhat negatively related to depressive symptoms [37, 50], whereas marital conflicts were related to higher depressive symptomatology [75, 76], therapeutic strategies could be examined to cope with (cultural) differences and inherent stress in marriage and other intimate relationships.

#### Ethnocultural factors

Turkish and Moroccan ethnicity appeared related to higher depression severity [37, 43, 51, 53, 71]. Older Moroccan-Dutch adults appeared as a vulnerable population [37] that might need more support to lead a healthy and functional life [77]. Furthermore, interventions aimed at improvement of the social network [76], social support (including same-ethnicity contacts for Turkish populations; [49, 63, 81]) might be a valuable addition to evidence-based treatments. Also, examining how patients could include helpful religious aspects in their daily life could be important, especially for Moroccan-Dutch [44, 81].

#### Acculturation

Depression treatment and prevention might benefit from reinforcing (Turkish) immigrant patients’ skills and social participation in the receiving society [46, 50, 54, 62, 86], while also reinforcing their ethnocultural social network [42, 82] Especially certain groups of patients might need more support developing an orientation to the receiving culture but might also profit the most of it: first generation patients, lower receiving-country language proficiency, lower education level, lower income, older age [46, 50, 56, 85], and lower emotional intelligence [62].

#### Discrimination

Evidence stemming from community samples showed that the negative impact of perceived ethnic discrimination on depression (prevalence) was strong [38, 42, 70, 73, 74, 82]. Clinical and, more importantly, social efforts to reduce or eradicate discrimination could contribute to the prevention and/or recovery of depression among Turkish and Moroccan immigrant populations (e.g., [18, 82]). Factors that appeared to weaken the association between perceived ethnic discrimination and depression were: a strong ethnic identity [73], emotional intelligence [62], practicing religion (for Moroccan individuals; [82]), and a same-ethnic network (for Turkish individuals; [82]). These aspects could be reinforced in therapeutic contacts.

### Strengths, limitations and research recommendations

To our knowledge, this is the first review addressing depressive symptoms and disorders of large (Turkish and Moroccan) immigrant populations in Northwestern Europe, with explicit attention for intersectionality, meaning the combination of psychological, sociodemographic, and ethnocultural factors that point towards more vulnerable as well as resilient subgroups within these populations. We excluded studies analyzing Turkish and Moroccan individuals together, which is an understandable practice to increase statistical power, but the presumption that both groups behave similarly was shown in this review to be invalid. Due to our method, that also evaluated the quality of the studies per ethnic group, we could compare these groups with similar migration history and highlight their uniqueness. Furthermore, we reviewed the literature systematically, and we assessed the methodological quality of all included papers with a standardized checklist of quality criteria predefined by the authors. Finally, this review also provided potential interventions for clinical practice based on the discussed findings.

This review knows some limitations. Despite the open language search strategy, no relevant papers from Spain, France or Italy, countries with a large Moroccan immigrant population, were retrieved by the search. This aspect limited the conclusions applicable to whole Europe and changed the focus of our paper to Northwestern Europe only. Furthermore, only a few studies were comparable based on the type of prevalence, the instruments used, and the population assessed, which only allowed a descriptive method to evaluate the (pooled) prevalence. Additionally, important topics fell out of the scope of the current review, such as depression related to somatic illnesses, suicide ideations, and a more broadly discussion of comorbid disorders and symptoms like anxiety and post-traumatic stress disorder, which are possibly also relevant for an adequate estimation of prevalence figures and mental health care aspects in these groups [13]. Also, none of the included studies differentiated between immigrant populations and self-defined ethnicity (i.e., self-report on sense of belonging to the receiving or the background culture; [106]), even though prevalence figures have been found to differ between these two groups in Europe [6]. We neither included sociological studies examining the relationship of social inequities with power dynamics at the individual, institutional, global, or socio-historical levels, which is an important anchor of intersectional theory [28]. Furthermore, only few studies examined mediating or moderation effects of the psychological, socio-demographic, and ethnocultural factors in relation to depressive symptomatology, which limited the conclusions on the intersectional level. In addition, we cannot draw conclusions about causality due to the cross-sectional design of the included studies.

In light of the above-mentioned results, strengths and limitations, some recommendations for future research are possible. First, future research should extend the cross-cultural psychometric knowledge of instruments used to measure depressive symptoms, as well as related constructs. A mutual agreement on the conceptualization of these constructs, e.g., acculturation strategies, would be helpful. Second, larger external validity and replicability of the findings should be sought, by including second-generation individuals, considering self-defined ethnicity, mid- highly educated and sexual minority individuals (concerning whom there is currently a research gap). Doing so, ecological validity and avoiding over-controlling for socio-demographic factors, e.g., income, education level, unemployment, is important. These factors reflect these immigrant populations’ reality and are likely to be important mediators or moderators in the association between ethnicity and depressive symptoms, though conclusive remarks cannot be made at this point. Therefore, the psychological, sociodemographic and ethnocultural factors should be taken into account in future intersectionality research of interlocking social identities, with more advanced statistical methods, like multilevel models [107]. Third, extensive and longitudinal clinical studies, like the NESDA (Netherlands Study of Depression and Anxiety; [108]), but also among immigrant populations, are needed to shed more light about the (individual) psychological, social, biological, genetic factors, and underlying mechanisms affecting the development and the long-term prognosis of anxiety and depression. Finally, some of the discussed findings point towards possible specific therapeutic interventions for Turkish and Moroccan immigrant populations with depressive disorders. These interventions should still be carefully tested for its added value to already existing evidence-based therapies.

To conclude, the mental health of Turkish and Moroccan immigrants with depression has received considerable attention in Northwestern Europe in the last two decades, in particular targeted at the Turkish groups in Germany, the Netherlands, and Belgium. Prevalence of depression in Turkish immigrants was found to be high, whilst in most Moroccan populations, it was rather similar to the prevalence of natives. Ethnic background and perceived ethnic discrimination were factors with salient links with higher depressive symptomatology in both groups. Future research is still highly warranted in order to achieve evidence-based diagnostics and treatments for these groups. Future studies should in particular target cross-cultural validity of used instruments, replicability of the findings in larger, more representative samples, and longitudinal and evaluative examination of mediating and moderating factors and effects of specific therapeutic interventions.

## Supplementary Information

**Additional file 1.**

**Additional file 2.**

**Additional file 3.**

**Additional file 4.**

## Data Availability

The present study is a systematic literature review. No individual data have been analyzed. All data and material used are available in the supplementary material (Additional files 1, 2, 3 and 4).
