# Supplementary material for: Depression among Turkish and Moroccan immigrant populations in Northwestern Europe: a systematic review of prevalence and correlates
Source: BMC Psychiatry. 2023 Jun 5;23:402. doi: 10.1186/s12888-023-04819-4 (PMC10240804; doi:10.1186/s12888-023-04819-4)
Supplement: Supplementary file 1 — Additional file 1. [file 12888_2023_4819_MOESM1_ESM.docx]

Additional file 1

*Detailed search strategy (Pubmed)*

(((((Turk* OR Morocc* OR Kurd* OR Berber*[Title/Abstract])) AND (Europ* OR European union OR EU OR Western Europe OR North Europe OR United Kingdom OR England OR Scotland OR UK OR Wales OR British OR Scottish OR Netherlands OR Holland OR Dutch OR Belgi* OR France OR French OR Spain OR Spanish OR Portug* OR German* OR Austria* OR Switzerland OR Swiss OR Ital* OR Finland OR Finn OR Denmark OR Danish OR Norw* OR Swed*[Title/Abstract])) AND (immigrant OR migrant OR migration[Title/Abstract]) AND (depress* OR depression OR mood W/1 disorder OR affective W/1 disorder OR depressiv* OR somatoform W/1 disorder OR psychosomatic OR somati* OR pain OR depression NOT postpartum) AND (illness representation OR illness belief OR manifestation OR idiom W/2 distress OR prevalence OR risk factor OR determinant OR protective factor OR correlat* OR resilience OR help-seeking W/2 behavior OR therapeutic W/1 rapport OR acculturation OR treatment OR therapy OR treatment W/2 expectation OR perceived need OR mental W/2 healthcare OR dropout OR no-show OR attrition OR adherence OR quality life OR well being [Title/Abstract])) AND ("1970/01/01"[Date - Publication] : "2017/07/31"[Date – Publication])
