# Supplementary material for: Depression among Turkish and Moroccan immigrant populations in Northwestern Europe: a systematic review of prevalence and correlates
Source: BMC Psychiatry. 2023 Jun 5;23:402. doi: 10.1186/s12888-023-04819-4 (PMC10240804; doi:10.1186/s12888-023-04819-4)
Supplement: Supplementary file 2 — Additional file 2. [file 12888_2023_4819_MOESM2_ESM.docx]

Additional file 2

*Characteristics of the included studies (n=51) and assessed study quality*

| **Author, year,**  **Country of study** | **Design; population type** | **Study sample**  **(N; % female; ageM** ±**SD/(range))**  **(n; ethnicity, % female; ageM** ±**SD/(range))** | **Instrument to measure depression** | **Other instruments** | **Study quality rating T/M** |
| --- | --- | --- | --- | --- | --- |
| **Prevalence** |  |  |  |  |  |
| Bermejo et al., 2016*  Germany | Cross-sectional, community sample | 435, 55.5%, 54.7 ± 12.4  - 77 T, 51.9%, 53.1±11  - 67 Spanish immigrants, 49.3%, 56.6±11.3  - 95 Italian immigrants, 41.1%, 47, 51.4 ±13.5  - 196 German repatriates, %, 65.6, 56.3±12.4 | PHQ-D | GAD-7 | 3 / - |
| Beutel et al., 2016*  Germany | Cross-sectional, cohort study;  general population | 14,943, -, (35-74),  - 11418 nG, 49.3%, 55.5 ± 11.1  - 141 TG, 50.9%, 52.6 ± 10.6  - 295 Polish-German, 49.4%, 54.7 ± 11.1  - 282 Western countries immigrants  - 386 Middle and Southern European immigrants. | PHQ-8 | GAD-7  PHQ-panic module  Mini-Spin  DS-14 | 3 / - |
| Braam et al., 2010*^÷^  The Netherlands | Cross-sectional, general population sample,  Amsterdam | 776, 57%, 51 ± 14.8, (19-82)  - 309 nD, 59%, 54  - 180 MD, 46%, 49  - 202 TD, 59%, 47  - 85 SAD, 72%, 52 | SCL-90-R (depression subscale)  CIDI 2.1, (section E) | brief RCOPE | 1 / 1 |
| Erim et al., 2011a  Germany | Cross-sectional,  clinical sample, outpatients with psychosomatic complaints | 51 TG, 78.4%, 39.41 ± 9.85 | SCID-I (296.2, 296.3, 300.4)  BDI | SOMS  ETI | 3 / - |
| Fassaert et al., 2010a  The Netherlands | Cross-sectional, general practice sample | 147,109, not stated, 51.8 ± 18.5  - 4884 TD; 72.5%, 38.7 ± 11.3  - 3458 MD; 67.1%, 35.7 ± 9.3  - 131,690 nD, 69.1%, 53.2 ± 18.6 | ICPC diagnosis (P03, P76) |  | 2 / 2 |
| Fassaert et al., 2010b  The Netherlands | Cross-sectional, clinical sample, in-outpatients | 17,270 episodes of treatment; (18-65)  - 947 TD, 68.7% 35.4 ± 8.3  - 834 MD, 58.4% 35.3 ± 8.7  - 12,824 nD, 65.4% 40.6 ± 11.7,  - Dutch Antillean  - Surinamese  - Other non-western  - Other western | DSM-IV-diagnosis (codes 296.21–296.24 and 296.31–296.34) |  | 2 / 2 |
| Fassbender and Leyendecker, 2018*  Germany | Cross-sectional, community sample | 327 T, 100%, 35.9 ±5.6 (24-59) | CES-D-10 | SWLS  Hassles Scale | 3 / - |
| Galenkamp et al., 2017*^╤^  The Netherlands | Cross-sectional, community sample, Amsterdam | 23,182, -  - 4028 TD, 54.9%, 44.2±11.5  - 4292 MD, 62%, 43.8±13.4  - 2444 Dutch-Ghanaians, 61.4%, 39.7 ±13.0  - 4428 Dutch-African Surinamese, 59.6%, 39.9 ±12.5  - 3355 Dutch-South-Asian Surinamese, 53.6%, 46.5±13.2  - 4635 nD, 54.1%, 46.2 ±14 | PHQ-9 |  | 2 / 2 |
| Ikram et al., 2015*^╤^  Netherlands | Cross-sectional, community sample, Amsterdam | 44.9%, (18-70)  - 834 TD, 53.2%, 39.8 ± 11.17  - 1744 nD, 53.5%, 46.5 ± 13.9  - 1126 South Asian Surinamese, 52.7%, 45.8 ± 13.14  - 1770 African Surinamese,62.1%; 46.8 ± 12.8  - 1072 Ghanaians-Dutch, 59.0%, 45.0 ± 12.2 | PHQ-9 | EDS | 1 / - |
| Janssen-Kallenberg et al., 2017  Germany | Cross-sectional, general population sample | 662 T/TG, 58.3%, 18-65 | CIDI 2.8 (section E) |  | 2 / - |
| Levecque et al., 2009*  Belgium | Cross-sectional, general population | 506, not stated, (18-65)  - 11747 nB; 52.0%, 41.98 ± 13.12  - 147 TB, 45.6%, 36.81 ± 10.24  - 359 MB; 48.2%, 38.74 ± 10.89 | GHQ-12  SCL-90-R  (depression, generalized anxiety) | MOSS | 1 / 2 |
| Morawa & Erim, 2014a*^╡^  Germany | Cross-sectional, community, convenience sample | 218, 67%, 40.0 ± 13.2  - 109 TG, 67%  - 109 PG, 67% | BDI | SF-36  Perceived discrimination Scale | 3 / - |
| Morawa et al., 2020*  Germany | Cross-sectional, general population | 328, 61.3%, 41.6 ± 11.3 (20-69) | PHQ-9 | FRACC  Life satisfaction (1 item) | 2 / - |
| Nieuwenhuijsen et al., 2015*^╤^  The Netherlands | Cross-sectional, community sample, Amsterdam | 6278, 50%, 43±12  1,355 Dutch  1,290 African Surinamese  1,121 South-Asian Surinamese  1,090 Turkish  729 Ghanaian  693 Moroccan | PHQ-9 | SF-12 (Mental Component Summary  Score).  Prolonged fatigue (1item).  Indicators of unfavorable working conditions & perceived stress at work. | 3 / 3 |
| Reich et al., 2018*^▲^  Germany | Cross-sectional, general population (“study 1”), convenience sample (“study 2”), inpatients (“study 3” & “study 4”) | 1977, 56.2%, 42.6 ±12.8  - 116 T (completing the Turkish version of PHQ-9): 61.2%, 43.7±11.1  - 191 T/TG (completing the German version of PHQ-9): 57.4%, 32.6±9.9  - 1670 nG: 55.7%, 43.7 ±12 | PHQ-9 | Migration-related characteristics | 2 / - |
| Sariaslan et al., 2014*^╡^  Germany | Cross-sectional, general practice sample | 418, 47.1%  - 254 TG, 42.9%, 38.37 ± 12.28  - 164 nD, 53.7%, 54.30 ± 18.34 | BDI | SOMS | 1 / - |
| Schrier et al., 2010^÷^  The Netherlands | Cross-sectional study, community sample, Amsterdam, random stratified sample | 812, -  - 213 TD, 60.1%, 47.3 ± 14.2  - 191 MD, 47.1%, 49.6 ± 14.4  - 321 nD, 58.3%, 54.1 ± 14.6  - 87 Surinamese-Dutch (SD), 71.3%, 52.3 ± 15.2 | CIDI 2.1 (section E)  SCL-90-R (depression) | WHODAS II | 2 / 2 |
| Schrier et al., 2012^÷^  The Netherlands | Cross-sectional study, community sample, Amsterdam | 698, -  - 205 TD, 61.5%, 47.5 ± 14.1  - 186 MD, 47.8%, 49.5 ± 14.5  - 307 nD, 58.6%, 54.1 ± 14.8 | CIDI 2.1 (sections D, E) | K10 | 2 / 3 |
| Snijder et al., 2017^╤^  The Netherlands | Cross-sectional study, community sample, Amsterdam | - 4200 TD, 54.3%, 39.9±12.5  - 4502 MD, 61.9%, 39.7±13.1  - 2735 Dutch-Ghanaians, 61.1%, 44.0±11.7  - 4458 Dutch-African Surinamese, 59.5%, 47.6±12.8  - 3369 Dutch-South-Asian Surinamese, 53.7%, 45.1±13.5  - 4671 nD, 54.1%, 46.1±14.1 | PHQ-9 | Multiple  instruments on  physical and  mental health  (presented in  the framework  of a study  protocol) | 3 / 3 |
| Stronks et al., 2020*^╤^  The Netherlands | Cross-sectional study, community sample, Amsterdam | 19,904 18-70  - 3800 TD  - 4088 MD  - 4212 Dutch-African Surinamese  - 3213 Dutch-South-Asian Surinamese  - 4591 nD | PHQ-9 | Everyday  Discrimination  Scale (EDS)  Sociocultural  conditions  (including  aspects such as  acculturation,  integration,  assimilation,  separation,  marginalisation,  ethnic identity,  ethnic social  network  composition)  PAS | 2 / 2 |
| Ünlü Ince et al., 2014*^÷^  The Netherlands | Cross-sectional, community sample, Amsterdam | 210 TD, 40%, 47.4 ± 14.2) | CIDI 2.1 (section D, E) | LAS | 2 / - |
| De Wit et al., 2008*^÷^  The Netherlands | Cross-sectional study, community sample, Amsterdam | 812, -, (19-92)  - 213 TD, 60%  - 191 MD, 47%  - 320 nD, 58%  - 88 Surinamese-Dutch (SD), 71% | CIDI 2.1 (sections D, E) | K10  MH15 | 2 / 2 |
| Van der Wurff et al., 2004*  The Netherlands | Cross-sectional study, non-institutionalized community sample | 933, 49.9%, 64.6 ± 5.3 (55-74)  - 330 TD, 50.3%, 63.5 ± 5.0  - 299 MD, 43.8%, 64.9 ± 4.8  - 304 nD, 55.6%, 65.4 ± 5.9 | CES-D | Ethnic-cultural identity scale (Martens, 1999; van den Reek, 1998; Kemper,  1996) | 1 / 1 |
| **Correlates** |  |  |  |  |  |
| Akbiyik et al., 2008  Germany, Turkey | Cross-sectional study, clinical sample, outpatients | 105, 44.9%  - 53 TG, 64%, 49,4 ± 8,4  - 52 nT, 73%, 44.7 ±9.2 | MINI  SCL-90-R  BDI | MANSA | 2 / - |
| Arens et al., 2013^║^  Germany | Cross-sectional study,  healthy and clinical sample | 108, 100%,  - 28 healthy TG (hTG), 100%, 43.6 ± 9.6  - 26 healthy nG (hnG), 100%, 43.8 ± 11.2  - 29 TG with depression (TGd), 100% 44.4 ±8.1  - 25 nG with depression (nGd), 100%, 43.4 ±10.7 | SCID-I  SCL-90-R  BDI | SCID-II  PANAS  ERQ  DAS-A | 3 / - |
| Balkir et al., 2013^║^  Germany | Cross-sectional design, clinical sample, inpatients | 56, 100%  - 29 TG, 100%, 44.5 ± 1.8  - 27 nG, 100%, 43.3 ± 1.9 | SCID-I  SCL-90-R (depression subscale, Global Severity Index) | SCID-II  PANAS  SCS | 3 / - |
| Balkir et al., 2013^║^  Germany | Cross-sectional design, clinical sample, inpatients | 110, 100%,  - 28 healthy TG, 100%, 43.6 ± 1.9  - 26 healthy nG, 100%, 43.9 ± 2.0  - 29 TG with depression (TGd),  100%, 43.3 ±1.9  - 27 nG with depression (nGd),  100%, 44.5 ±1.8 | SCID-I  SCL-90-R (depression subscale, Global Severity Index) | SCID-II  PANAS  Loneliness Scale,  Basic Psychological Need Satisfaction Scale (autonomy and relatedness subscales) | 3 / - |
| Baltas and Steptoe, 2000  United Kingdom | Cross-sectional study, community sample | 66, 50%, 39.3 ± 9.2, (26-54)  - 33 TBr, 30.3%  - 33 nBr, 69.7% | BDI | STAI (trait subscale)  Marital cultural difficulties index | 3 / - |
| Bengi-Arslan et al., 2002  The Netherlands | Cross-sectional,  general population | 785 TD, 66.6%, age not mentioned | GHQ-28 (severe depression) | Turkish Immigrant Assessment Questionnaire | 2 / - |
| Brandl et al., 2020 | Cross-sectional study, clinical sample, outpatients | 381, 58.8% women, 43.9 ± 12.5,  (18-70)  - 111 TG, 76.6%, 45.3 ±10.9  - 39 sample with East European migration background, 53.9%, 43.2 ± 13.2  - 37 sample with a MENAP (Middle East, North Africa, Afghanistan/Pakistan) migration background, 48.6%, 38.2 ± 10.5  - 194 sample without a migration background, 51.6%; 44.3 (±13.4) | SCL-14 | Questionnaire on Attitudes Toward Psychotherapeutic  Treatment (QAPT)  Drug Attitude Inventory (DAI)  Acculturation Index | 2 / - |
| Erim et al., 2011b  Germany | Cross-sectional study, clinical sample, outpatients | 156, 76.9%, 37.4 (± 9.78, 19-71)  - 96 TG, 76%, 36.67 (±9.52)  - 60 nT, 78.3%, 38.57 (±10.15)  - German general population norm value  - German outpatient population norm value | ICD-10 diagnosis (codes F3, F4, F5)  BDI | SOC-29 | 2 / - |
| Gül and Kolb, 2009  Germany | Cross-sectional study, clinical sample, outpatients | 220 TG, 42.9%, 23.4 ± 3.49, (18-30)   - 154 well acculturated TG - 66 marginalized / separated TG | ICD-10 diagnosis (code F1, F2, F42, F32, F41, F42, F43.2) | 5-item Acculturation Questionnaire | 3 / - |
| Gündüz et al., 2018  Austria | Cross-sectional, community sample and  clinical sample | 297, -  - 144 patients with depression (Turkish people in homeland: 49, 75.5%, 36.4±9.4; Turkish immigrants in Austria: 48 60.4% 44.3±9; Austrians: 47, 63.8%, 45.4±8.1)  - 153 control subjects (Turkish people in homeland: 51, 7.05%, 29.8±12; Turkish immigrants in Austria: 53, 56.6%, 33.9±9; Austrians: 49, 40.8%, 30.8±11.9) | ICD-10 diagnosis | RSQ | 3 / - |
| Ikram et al., 2016^╤^  The Netherlands | Cross-sectional study, community sample,  Amsterdam | 11780, 58.4% women, 43.0 ± 13.1,  (18-70)  - 2484 MD, 62.5%, 39.3 ± 13.1  2626 TD, 54.3%; 39.8 ± 12.4  - 2501 South Asian Surinamese, 54.3%, 45.3 ±13.4  - 2292 African Surinamese,62.7%, 46.9 ± 12.8  - 1877 Ghanaians-Dutch, 59.1%; 44.6 (±11.4) | PHQ-9 | EDS  Psychological Acculturation Scale (ethnic identity subscale)  Ethnic social network (2 questions) | 1 / 1 |
| Kizilhan et al., 2015  Germany | Cross-sectional,  clinical sample, inpatients with psychosomatic complaints | 270 TG, 100%, (30-50)  - 120 TG forced to marry, 42.9%  - 150 TG not forced to marry, 46.7% | BDI  SCL-90-R (depression) | Koch’s sociodemographic questionnaire (1997) | 3 / - |
| Mewes et al., 2010  Germany | Cross-sectional, general population sample | 134, -  - 42 TG, 31%, 30.9±10.5  - 43 East European-German (EeG), 67%, 51.7±21.5  - 49 Soviet Union-German (SUG), 53%, 44.3±19.6 | PHQ-9 | PHQ-15, PHQ-general anxiety | 3 / - |
| Mewes et al., 2015^▲^  Germany | Cross-sectional, general population sample | 214 TG, 63%, 33 ± 10.9, (18-66) | PHQ-9 | PHQ-15  PSS-10  BIAS-TS  IPAC | 2 / - |
| Morawa & Erim, 2014b  Germany | Cross-sectional, clinical sample, out- and inpatients with psychosomatic complaints | 471 TG, 46.3%, 39.7 ± 11.5 | BDI | FRACC | 2 / - |
| Müller et al., 2017  Germany | Cross-sectional, clinical sample | Two samples:  Sample 1: 98, 65.3%, 40.4 ± 11.6  - 48 TG, 60.4%, 43.4 ± 10.8  - 50 nG, 70%, 37.5 ± 11.6  Sample 2: 749, 55.5%, 46.8 ± 18.3  - 187 people with migration background (including 90 from Turkey or Arabic countries), 61.5%, 43.2 ± 16.1  - 562 nG, 53.5%, 48.0 ± 18.8 | DSM-IV-TR (text revision) and ICD-10  criteria | MIGSTR10 | 3 / - |
| Nap et al., 2015  The Netherlands | Cross-sectional;  naturalistic, longitudinal (not the reviewed section), clinical sample, outpatients | 737, -  - 197 TD, 60.4%, median = 37  - 328 MD, 48.8%, median =35  - 212 SD, 70.6%, median = 40 | BSI (depression subscale) | BSI (somatization, anxiety, depression subscale)  LAS  EQ-5D  Patient Request Form (PBV) Dutch shortened version | 2 / 2 |
| Reijneveld et al., 2007  The Netherlands | Cross-sectional, community sample | 933, 49.9%, 64.6 ± 5.3, (55-74)  - 330 TD, 50.3%, 63.5 ± 5.0  - 299 MD, 43.8%, 64.9 ± 4.8  - 304 nD, 55.6%, 65.4 ± 5.9 | DSM-IV-diagnosis,  CES-D | Katz’questionnaire  SF-36  OECD mobility scale | 2 / 2 |
| Sanchez-Teruel & Robles-Bello, 2020  Spain | Cross-sectional, community sample | 326, 0%, 19.6 ± 1.13, (18-23)  - 154 MSp, 0%, 19.3 ± 1.28  - 172 non-immigrant Spanish men, 0%, 20.1 ± 1.06 | BDI-II | STAI  Life-Orientation Test (LOT)  Herth Hope Index (HHI) Herth  General Self-Efficacy Scale (GSE)  Multidimensional Scale of Perceived Social Support  (MSPSS)  14-Item Resilience Scale (RS-14) | - / 2 |
| Schrier et al., 2013^÷^  The Netherlands | Cross-sectional study, community sample, Amsterdam | 682, 44.9%  - 203 TD, 61.1%, 44.7 ± 9.2  - 170 MD, 42.9%, 49.4 ± 14.6  - 309 nD, 58.9%, 54.1 ± 14.7 | CIDI 2.1. (section D, E)  SCL-90-R (depression subscale) | NEO-FFI SCL-90-R (anxiety, phobic anxiety subscales) | 2 / 2 |
| Schmitz & Schmitz, 2012  Germany | Cross-sectional study,  community sample | N1= 349, 48.2%  - 197 TG, 48.2%, 22.8  - 150 North-African-German (e.g., Moroccan, Algerian, Tunisian) (NAG), 58%, 23.4  N2 = 65, 61.5%, 21.3  - 44 TG  - 21 North-African-German (NAG) | BDI | TMMS-24  AAS  SWLS  SHS  Immigrant Adolescent Questionnaire (unfairness scale)  Acculturation Behavior Ratings | 3 / - |
| Selten et al., 2012  The Netherlands | Cross-sectional study, clinical sample, in- and outpatients, Utrecht | 862762 case files, 50.1%, (15-65)  - 41226 TD, 48.7%  - 72484 MD, 47.3%  - 692132 nD, 51.6%  - 26345 SD, 51.4%  - 30575 Western-European (WE), 51.6% | DSM-IV-diagnosis (codes 296.2x, 296.3x, 296.0, 296.4x, 296.5x,  296.6x,  296.7x,  296.89,  295.x,  297.1, 298.8,  298.9) |  | 3 / 3 |
| Slotman et al., 2017^╤^  The Netherlands | Cross-sectional study, community sample, Amsterdam | 9141, -  - 2204 TD, 53.6%, 39.9±12.4  - 1476 MD, 56.9%, 38.8±12.9  - 1355 Dutch-Ghanaians, 58.1%, 44.8 ±12.2  - 4156 Dutch-Surinamese, 58.4%, 46.2 ±13 | PHQ-9 | EDS (adapted  version)  PMS | 1 / 1 |
| Szabó, et al., 2020^≠^  The Netherlands | Cross-sectional study,  general population | 478, 42%, 60.9±3, 55-65  - 268 TD, 45%, 60.8±3.1  - 209 MD, 39%, 61.1±2.9 | 20-item  Center for Epidemiologic Studies Depression scale | The Pearlin  Mastery  Scale  De Jong  Gierveld  Loneliness  Scale  Items on  religious  coping,  language  proficiency,  closeness with  Dutch, feelings  of loss,  participation in  Dutch  organizations,  cultural  identity  exploration and sociodemographic characteristics | 1 / 1 |
| Tagay et al., 2008  Germany | Cross-sectional study,  clinical sample,  general practice patients | 195 Turkish/Kurdish German, 63.6%, 40.5 ± 13.3 | HADS | ETI | 3 / - |
| Uslucan, 2005  Germany | Cross-sectional study, convenience sample (general practice patients and community sample), Berlin | 357 TG, 63.6%, 34.3 ± 12.3 (13-66) | ICD-10 screening (depressive symptoms) | Dundee Relocation Inventory  Self-esteem Scale  Emotional avoidance coping  Social Support Scale | 3 / - |
| Van Dijk et al., 2010  The Netherlands | Cross-sectional study, community sample, Amsterdam | 352, 49%, 19 (15-24)  - 199 TD, 44.7%, 18.9 (±2.7)  - 153 MD, 54.9%, 18.6 (±2.7) | CES-D | Perceived discrimination scale | 1 / 1 |
| Van Tilburg and Fokkema, 2020^≠^  The Netherlands | Cross-sectional study,  general population | 703, 55-66  - 235 TD, 60.8 ±3.1  - 176 MD, 60.9±2.9  - 292 nG, 60.6±3.1 | CES-D | De Jong  Gierveld  Loneliness  Scale  The Pearlin  Mastery  Scale  Mini-Mental State Examination  Items on social and socio-demographic aspects  Items on chronic physical health problems | 1 / 1 |

Note: *Study also belongs to the ‘correlates’ section.

^╤^ Studies based on the same sample/cohort.

^╡^ Studies based on the same sample/cohort.

^▲^ Studies based on the same sample/cohort.

^÷^ Studies based on the same sample/cohort.

^║^Studies based on the same sample/cohort.

^≠^ Studies based on the same sample/cohort.

Abbreviations: T = Turkish sample, M = Moroccan sample; Study quality rating 1 = strong quality (SQ), 2 = moderate quality (MQ), 3 = weak quality (WQ); TD = Turkish-Dutch; MD = Moroccan-Dutch; nD = native-born Dutch; TB = Turkish-Belgian; MB = Moroccan-Belgian; nB = native-born Belgian; TG = Turkish-German; nG = native-born German; TBr = Turkish-British; nBr = native-born British; MSp= Moroccan-Spanish. Instruments abbreviations: AAS = Acculturation Attitude Scale; BDI = Beck Depression Inventory; BIAS-TS = Behaviors from Intergroup Affects and Stereotypes–Treatment Scale; Brief RCOPE = Short measure of religious coping; BSI = Bradford Somatic Inventory; CES-D = Center for Epidemiologic Studies Depression Scale; CIDI = Composite International Diagnostic Interview; DAS-A = Dysfunctional Attitude Scale Form A; DS-14 = Type D Scale-14; EQ-5D = EuroQol five dimensions questionnaire; EDS = Everyday Discrimination Scale; ERQ = Emotion Regulation Questionnaire; ETI = Essen Trauma Inventory; FRACC = Frankfurt Acculturation Scale; GAD-7 = Generalized Anxiety Disorder -7; GHQ-28, 12 = General Health Questionnaire 28, 12; HADS = Hospital Anxiety and Depression Scale; ICD-10 = 10^th^ International Classification of Diseases and Related Health Problems; ICPC = International Classification of Primary Care; IPAC = International Physical Activity Questionnaire; K10 = Kessler Psychological Distress Scale; LAS = Lowlands Acculturation Scale; MANSA = Manchester Short Assessment of Quality of Life; MH15 = Mental Health Indicator; MIGSTR10 = Questionnaire for Clinician-Based Assessment of Migration-Related Stressors; MINI = Mini International Neuro-psychiatric Interview; Mini-Spin = Mini-Social Phobia Inventory; MOSS = Medical Outcome Social Support Scale; NEO-FFI = NEO Five Factor Inventory; OECD mobility scale = Long-term limitations in mobility; PANAS = Positive and Negative Affect Schedule; PHQ-9, 15 = Patient Health Questionnaire-9, 15; PMS = Pearlin-Schooler Mastery Scale; PSS-10 = Perceived Stress Scale; RSQ = Relationship Scales Questionnaire; SCID-I = Structured Clinical Interview for DSM-IV axis I; SCID-II = Structured Clinical Interview for DSM-IV axis II SCL-90-R = Symptom Checklist-90-Revised; SCS = Self-Construal Scale; SF-12 = 12-Item Short Form Health Survey Questionnaire; SF-36 = 36-Item Short Form Health Survey Questionnaire; SHS = Subjective-Happiness-Scale; SOC-29 = Sense of Coherence scale; STAI = State – trait Inventory Anxiety; SWLS = Satisfaction with Life Scale; TMMS-24 = Trait-Meta Mood-Scale; WHODAS II = World Health Organization Disability Assessment Schedule II.
