# Supplementary material for: Depression among Turkish and Moroccan immigrant populations in Northwestern Europe: a systematic review of prevalence and correlates
Source: BMC Psychiatry. 2023 Jun 5;23:402. doi: 10.1186/s12888-023-04819-4 (PMC10240804; doi:10.1186/s12888-023-04819-4)
Supplement: Supplementary file 3 — Additional file 3. [file 12888_2023_4819_MOESM3_ESM.docx]

Additional file 3
*Criteria for quality assessment of included studies*

| *Criteria for quantitative studies* | Scoring per criteria | Scoring per section  (separate scoring for Turkish and Moroccan sample)  (1) strong; (2) moderate; (3) weak; (NA) not applicable |
| --- | --- | --- |
| 1. **Selection bias** | | |
| a. Representativity of the specific target group sample^a^ | 1. Very likely (randomly selected)  2. Somewhat likely (referred from a source e.g. clinic in a systematic manner)  3. Not likely (self-referred)  4. Can’t tell | Sum of all items  1.Strong: 5- 6  2. Moderate: 7- 12  3. Weak: 13+ |
| b. Response rate ^a^ | 1. 80 - 100% response  2. 60 – 79% response  3. less than 60% response  4.Can’t tell |  |
| c. Both male and female participants^b^ | 1. (Fairly) equal proportion of men and women (50%, +-20%)  2. Greatly uneven proportion of men and women  3. Only men or women |  |
| d. Detailed description of the sample^b^ | 1. Description of basic elements (gender, age, ethnicity) and 2 of more socio-demographic, socio-economic indicators  2. Description of basic elements (gender, age, ethnicity) and one extra socio-demographic or socio-economic indicator  3. Description of only basic elements (gender, age, ethnicity) or no description |  |
| e. Inclusion/ exclusion criteria clearly stated | 1. Yes (both inclusion and exclusion criteria are stated)  2. Partially (only inclusion or exclusion criteria are stated)  3. No |  |
| **2. Study design** | | |
| a. Type of design^a^ (control group?) | 1.Randomized controlled trial (RCT)  1. Contro1lled clinical trial (Cct)  2. Cohort analytic (two group pre + post)  2. Case-control intervention study/ between-groups cross-sectional  2. Cohort (one group pre + post (before and after))  2. Interrupted time series  3. Single-subject cross-sectional  3. Other  3. Can’t tell | 1.Strong:  a = 1 with b or c= 1 or 2 (then they are seen as Cct)  2.Moderate: a=2  3.Weak: a=3 |
| b. Randomization method described (only for RCT)^a^ | 1. Yes  2. No  NA. Not applicable |  |
| c. Randomization method appropriate (only for RCT)^a^ | 1.Yes  2.No  NA. Not applicable/ can’t tell |  |
| **3. Confounders** | | |
| 1. Interventions: Important differences between groups at baseline (ethnicity, sex, marital status, age, SES, education, etc.)^a^   Cross-sectional study: Possible confounders responsible for the associations (mentioned by the authors) | 1.Yes  2. No  3. Can’t tell | 1.Strong: a = 1 and b = 1 or a = 2  2.Moderate: a=1 and b = 2  3. Weak: a = 1 and b = 3 or 4, a = 3 |
| b. Percentage confounders /difference that were controlled for (i.e. stratification, matching, as covariates in analyses)^a^ | 1. 80 – 100% (most) / Not applicable (No important differences)  2. 60 – 79% (some)  3. Less than 60% (few or none)  4. Can’t Tell |  |
| **4. Blinding (RCT’S, controlled clinical trials)** | | |
| a. Assessors were aware of the intervention^a^ | 1.No  2.Yes  3.Can’t tell  NA. Not applicable | 1.Strong: a = 1 and b = 1  2.Moderate:  a = 2/3 and b = 1  a = 1 and b = 2/3  3. Weak: a = 2 and b = 2  a = 3 and b is 3  NA. data was self-reported or collected by surveys, questionnaires or interviews |
| b. Participants were aware of the intervention^a^ | 1.No  2.Yes  3.Can’t tell  NA. Not applicable |  |
| **5. Data collection methods** | | |
| a. Valid (assess construct accurately) instruments for the specific target group (T/M)^a^ | 1.Yes, totally (all of the relevant instruments, validated in T/M migrant samples)  2. Yes, partially (not all the relevant instruments used are valid, or validated in other migrant samples, or provided back-translated instruments)  3.No  4.Can’t tell  Read relevant as needed for our purpose (depression instrument and other related instruments) | 1.Strong:  a = 1 and b = 1  2. Moderate:  a = 1 and b = 2, 3 or 4  a = 2 and b = 1 or 2  a = 3 or 4 and b = 1    3. Weak:  a = 2 and b = 3 or 4  a = 3 or 4 and b = 2, 3 or 4 |
| b. Reliable (internally consistent) instruments for the specific target group (T/M)^a^ | 1.Yes, totally (all of the relevant instruments)  2. Yes, partially (not all the relevant instruments used are reliable)  3.No (as indicated by Cronbach alpha in the current study)  4.Can’t tell (no Cronbach alpha of the current study reported)  Read relevant as needed for our purpose (depression instrument and other related instruments) |  |
| **6. Withdrawal and drop-outs (interventions and longitudinal studies)** | | |
| a. Report of numbers and reasons of drop-out per group^a^ | 1. Yes (both numbers and reasons per group)  2. No  3. Can’t tell  NA. Not Applicable (i.e. one time surveys or interviews) | 1.Strong:  a=1 and b = 1  2. Moderate:  a= 1 and b = 2  3. Weak:  a = 2, 3;  a= 1 and b = 3  NA. Not Applicable |
| b. Percentage of people completing the study or included in the final analysis per group (rate lowest percentage)^a^ | 1. 80 -100%  2. 60 - 79%  3. less than 60%  4. Can’t tell  NA. Not Applicable (i.e. retrospective case-control) |  |
| **7. Intervention integrity** | | |
| a. Percentage of participants receiving the complete intervention (experimental completers of initial sample without follow-up)^a^ | 1. 80 -100%  2. 60 - 79%  3. less than 60%  4. Can’t tell  NA. Not Applicable (no intervention study) | 1.Strong:  a = 1 and b = 1 and c = 1  2. Moderate:  a = 1 and b = 2 and c = 1  a = 2 and b = 1 or c = 1  a = 2 and b = 2 and c = 1  3. Weak:  a = 3 and b = 1 and c = 1  a = 2 and b = 2/3 and c = 2/3  a = 3 / 4 and b = 2/3 or c = 2/3  Sum of all items  1.Strong: 3  2. Moderate: 4-5  3. Weak: 6+  NA. Not Applicable |
| b. Consistency of intervention^a^ | 1.Yes (a method to measure if same intervention was provided to all participants is described)  2. No  3.Can’t tell  NA. Not applicable |  |
| c. Robust intervention (contamination or co-intervention unlikely)^a^ | 1.Yes  2. No  3.Can’t tell  NA. Not applicable |  |
| **8. Analyses** | | |
| a. Sample size large enough to detect an effect of 5% or more in or between the groups^b^ | 1.Yes (50 per group)  2. Not likely / partially  3. No | Sum of all items (excluding c)  1.Strong: 5-7  2. Moderate: 8-10  3. Weak: 11+ |
| b. A priori sample size calculation described^b^ | 1.Yes  2. No |  |
| c. Unit of allocation (only RCT’s – unit randomized to the interventions, mostly individuals)^a^ | 1.community  2.organization/institution  3.practice/office  4.individual  NA. Not applicable (not an RCT) |  |
| d. Appropriate correspondence between research question (s), study design and statistical methods (i.e. intention to threat is appropriate) ^a,b^ | 1. Yes  2. Partially (not all analyses)  3. No |  |
| e. Effect sizes reported^b^ | 1.Yes  2. Partially (not for all analyses)  2. No |  |
| f. Use of imputation methods rather than actual data^b^ | 1. No missings, only completers (described)  2. Yes (imputation method or procedure in case of missings described)  3. Yes, incomplete information (missings are mentioned, but not a method to handle them)  4. Can’t tell (nothing described about missings) |  |
| **9. Global rating** |  |  |
|  |  | 1. STRONG (no WEAK ratings for any section)  2. MODERATE (one WEAK rating in one of the sections)  3. WEAK (two or more WEAK ratings across the sections) |
|  |  |  |
| ***Criteria for qualitative studies*** | ***Quality indicators (possible, not extensive, features for consideration)^d, e^*** | ***Scoring*** |
| 1.Research question clearly defined^c^ | a. Statement of why the research was done  b. Clear formulation of the specific question that is addressed | 1.Strong: Complete/ detailed/ clear information (a and b)  2.Moderate: Incomplete/ vague information (a or b)  3. Weak: Can’t tell/ Not described |
| 2.Appropriate use of a qualitative approach^d^ | a. The objective of the research was to explore, interpret, or obtain a deeper understanding of a particular clinical issue | 1. Strong: Yes  2. Moderate: Partially yes  3. Weak: No |
| 3.Context of research setting is clearly described^c,d^ | a. The (historical/social/organizational) setting in which the research is done is clearly described  b. The researchers’ perspective, vision, cultural background are described | 1. Strong: Complete/ detailed/ clear information (a and b)  2. Moderate: Incomplete information (a or b)  3. Weak: Vague information/ Can’t tell/ Not described |
| 4.Sampling strategy clearly described and justified^c,d^ | a. Description of population of interest  b. Rationale for basis of target sample  c. Description of methods of access and approach | 1. Strong: Information allows straightforward replicability  2. Moderate: Incomplete information  3. Weak: Vague information/ Can’t tell/ Not described |
| 5.Sampling strategy ensured generalisability^c^ | a. Profile of achieved sample allows making conclusions that concerns the whole population  b. Maximizing inclusion (e.g. language matching, specialized recruitment) | 1. Strong: Yes  2. Moderate: Moderately  3. Weak: No |
| 6. Data-collection procedure was clearly described^c,d^ | a. Discussion of who conducted the data collection.  b. Discussion of conventions for data-collection | 1. Strong: Information allows straightforward replicability  2. Moderate: Incomplete information  3. Weak: Vague information/ Can’t tell/ Not described |
| 7. Data analysis procedure clearly described and justified^c^ | a. Description of the form of original data (e.g. use of verbatim transcripts, etc)  b. The analysis related to the original research question  b. The method to identify themes and concepts was clear and justified | 1. Strong: Information allows straightforward replicability  2. Moderate: Incomplete information  3. Weak: Vague information/ Can’t tell/ Not described |
| 8. Evidence (citations) was used in the paper and available for independent analysis^c,d^ | a. The authors cite actual data.  b. The cited data is appropriate. | 1. Strong: 80 – 100% (in most of the statements)  2. Moderate: 60 – 79% (some)  3. Weak: Less than 60% (few or none) |
| 9. Reliability of analysis^c,d^ | a. The data analysis was done by many researchers. | 1. Strong: >2 researchers  2. Moderate: 2 researchers  3. Weak: One researcher/ Can’t tell/ Not described |
| 10. Diverse observations were taken into account^c^ | a. There was evidence of seeking out observations that might have contradicted/ modified the analysis  b. Evidence of attention to outliers, exceptions or negative cases.  c. Identification of patterns of associations with divergent position. | 1. Strong: Yes, many times (>4)  2. Moderate: Yes, sometimes (3-4)  3. Weak: Few or none (1-2) |
| 11. Link between data, interpretations and conclusions is logic, comprehensible^c,d^ | a. Interpretation is relatively untainted with personal perspective  b. The interpretation is a comprehensible result of the data analysis.  c. The interpretation is reasonably coherent with what is already known. | 1. Strong: 80 – 100% conclusions are comprehensible (most)  2. Moderate: 60 – 79% (some)  3. Weak: Less than 60% (few or none) |
| **Global rating^a^** |  | 1. STRONG (no WEAK ratings for any section)  2. MODERATE (one WEAK rating in one of the sections)  3. WEAK (two or more WEAK ratings across the sections) |

**^a^** Tool of the Effective Public Health Practice Project

**^b^** Added criteria relevant for current purpose

**^c^** BMJ Qualitative research checklist

^d^ Greenhalgh & Taylor, 1997

^e^ Quality in Qualitative Evaluation Framework
