# Supplementary material for: Depression among Turkish and Moroccan immigrant populations in Northwestern Europe: a systematic review of prevalence and correlates
Source: BMC Psychiatry. 2023 Jun 5;23:402. doi: 10.1186/s12888-023-04819-4 (PMC10240804; doi:10.1186/s12888-023-04819-4)
Supplement: Supplementary file 4 — Additional file 4. [file 12888_2023_4819_MOESM4_ESM.docx]

Additional file 4
*Detailed quality ratings of the included studies following rules in Appendix C*

| **Study, year** | Sample (T/M) | Quality Criteria | | | | | | | | | | | Global  quality rating | |  |
| --- | --- | --- | --- | --- | --- | --- | --- | --- | --- | --- | --- | --- | --- | --- | --- |
|  |  | Sum  Selection bias | 1. Selection bias | 2. Study design | 3. Confounders | 4. Blinding | 5.  Data collection | 6. Withdrawals dropouts | Sum Intervention integrity | 7. Intervention integrity | Sum  Analyses | 8. Analyses | |  | |
| Akbiyik et al., 2008 | T | 8 | 2 | 2 | 1 | na | 3 | na | na | na | 10 | 2 | | 2 | |
| Arens et al., 2013 | T | 13 | 3 | 2 | 1 | na | 2 | na | na | na | 11 | 3 | | 3 | |
| Balkir et al., 2013 | T | 13 | 3 | 2 | 1 | na | 2 | na | na | na | 13 | 3 | | 3 | |
| Balkir, et al., 2013 | T | 13 | 3 | 2 | 1 | na | 3 | na | na | na | 12 | 3 | | 3 | |
| Baltas & Steptoe, 2000 | T | 12 | 2 | 2 | 3 | na | 3 | na | na | na | 13 | 3 | | 3 | |
| Bengi-Arslan et al., 2002 | T | 11 | 2 | 3 | 1 | na | 1 | na | na | na | 6 | 1 | | 2 | |
| Bermejo et al., 2016 | T | 11 | 2 | 3 | 1 | na | 3 | na | na | na | 11 | 3 | | 3 | |
| Beutel et al., 2016 | T | 6 | 1 | 2 | 3 | na | 3 | na | na | na | 7 | 1 | | 3 | |
| Braam et al., 2010 | T | 8 | 2 | 2 | 1 | na | 2 | na | na | na | 10 | 2 | | 1 | |
| Braam et al., 2010 | M | 8 | 2 | 2 | 1 | na | 2 | na | na | na | 10 | 2 | | 1 | |
| Brandl et al., 2020 | T | 11 | 2 | 3 | 2 | na | 4 | na | na | na | 8 | 2 | | 2 | |
| De Wit et al., 2008 | T | 8 | 2 | 2 | 1 | na | 3 | na | na | na | 10 | 2 | | 2 | |
| De Wit et al., 2008 | M | 8 | 2 | 2 | 1 | na | 3 | na | na | na | 10 | 2 | | 2 | |
| Erim et al., 2011 | T | 7 | 2 | 3 | 3 | na | 2 | na | na | na | 11 | 3 | | 3 | |
| Erim et al., 2011 | T | 11 | 2 | 2 | 1 | na | 2 | na | na | na | 11 | 3 | | 2 | |
| Fassaert, Nielen et al., 2010 | T | 11 | 2 | 2 | 1 | na | 3 | na | na | na | 10 | 2 | | 2 | |
| Fassaert, Nielen et al., 2010 | M | 11 | 2 | 2 | 1 | na | 3 | na | na | na | 10 | 2 | | 2 | |
| Fassaert, Peen et al., 2010 | T | 11 | 2 | 2 | 1 | na | 3 | na | na | na | 7 | 1 | | 2 | |
| Fassaert, Peen et al., 2010 | M | 11 | 2 | 2 | 1 | na | 3 | na | na | na | 7 | 1 | | 2 | |
| Fassbender and Leyendecker, 2018 | T | 11 | 2 | 2 | 3 | na | 3 | na | na | na | 7 | 1 | | 3 | |
| Galenkamp et al., 2017 | T | 10 | 2 | 2 | 1 | na | 3 | na | na | na | 7 | 1 | | 2 | |
| Galenkamp et al., 2017 | M | 10 | 2 | 2 | 1 | na | 3 | na | na | na | 7 | 1 | | 2 | |
| Gül and Kolb, 2009 | T | 12 | 2 | 2 | 3 | na | 3 | na | na | na | 12 | 3 | | 3 | |
| Gunduz et al., 2018 | T | 9 | 2 | 3 | 1 | na | 3 | na | na | na | 11 | 3 | | 3 | |
| Ikram et al., 2015 | T | 8 | 2 | 2 | 1 | na | 2 | na | na | na | 9 | 2 | | 1 | |
| Ikram et al., 2016 | T | 8 | 2 | 2 | 1 | na | 2 | na | na | na | 8 | 2 | | 1 | |
| Ikram et al., 2016 | M | 8 | 2 | 2 | 1 | na | 2 | na | na | na | 8 | 2 | | 1 | |
| Janssen-Kallenberg et al., 2017 | T | 9 | 2 | 3 | 2 | na | 2 | na | na | na | 9 | 2 | | 2 | |
| Kizilhan et al., 2015 | T | 15 | 3 | 2 | 3 | na | 2 | na | na | na | 8 | 2 | | 3 | |
| Levecque et al., 2009 | T | 7 | 2 | 2 | 1 | na | 2 | na | na | na | 10 | 2 | | 1 | |
| Levecque et al., 2009 | M | 7 | 2 | 2 | 1 | na | 3 | na | na | na | 10 | 2 | | 2 | |
| Mewes et al., 2010 | T | 8 | 2 | 2 | 1 | na | 3 | na | na | na | 12 | 3 | | 3 | |
| Mewes et al., 2015 | T | 12 | 2 | 3 | 1 | na | 2 | na | na | na | 9 | 2 | | 2 | |
| Morawa, & Erim, 2014a | T | 6 | 1 | 3 | 1 | na | 2 | na | na | na | 8 | 2 | | 2 | |
| Morawa, & Erim, 2014b | T | 12 | 2 | 2 | 1 | na | 2 | na | na | na | 7 | 1 | | 1 | |
| Morawa et al., 2020 | T | 8 | 2 | 3 | 3 | na | 2 | na | na | na | 8 | 2 | | 2 | |
| Nap et al., 2015 | T | 12 | 2 | 2 | 3 | na | 2 | na | na | na | 10 | 2 | | 2 | |
| Nap et al., 2015 | M | 12 | 2 | 2 | 3 | na | 2 | na | na | na | 10 | 2 | | 2 | |
| Nieuwenhuijsen et al., 2015 | T | 8 | 2 | 2 | 3 | na | 3 | na | na | na | 8 | 2 | | 3 | |
| Nieuwenhuijsen et al., 2015 | M | 8 | 2 | 2 | 3 | na | 3 | na | na | na | 8 | 2 | | 3 | |
| Reich et al., 2018 | T | 10 | 2 | 2 | 1 | na | 3 | na | na | na | 8 | 2 | | 2 | |
| Reijneveld et al., 2007 | T | 6 | 1 | 2 | 3 | na | 2 | na | na | na | 8 | 2 | | 2 | |
| Reijneveld et al., 2007 | M | 6 | 1 | 2 | 3 | na | 2 | na | na | na | 8 | 2 | | 2 | |
| Sariaslan et al., 2014 | T | 7 | 2 | 2 | 1 | na | 2 | na | na | na | 9 | 2 | | 1 | |
| Sanchez & Robles, 2020 | M | 12 | 2 | 2 | 2 | 6 | 2 | na | na | na | 13 | 3 | | 3 | |
| Schrier et al., 2010 | T | 8 | 2 | 2 | 2 | na | 3 | na | na | na | 10 | 2 | | 2 | |
| Schrier et al. 2010 | M | 8 | 2 | 2 | 2 | na | 3 | na | na | na | 9 | 2 | | 2 | |
| Schrier et al., 2012 | T | 6 | 1 | 2 | 3 | na | 2 | na | na | na | 7 | 1 | | 2 | |
| Schrier et al., 2012 | M | 6 | 1 | 2 | 3 | na | 3 | na | na | na | 7 | 1 | | 3 | |
| Schrier et al., 2013 | T | 6 | 1 | 2 | 1 | na | 3 | na | na | na | 7 | 1 | | 2 | |
| Schrier et al., 2013 (49) | M | 6 | 1 | 2 | 1 | na | 3 | na | na | na | 7 | 1 | | 2 | |
| Selten et al., 2012 | T | 13 | 3 | 2 | 3 | na | 3 | na | na | na | 6 | 1 | | 3 | |
| Selten et al., 2012 | M | 13 | 3 | 2 | 3 | na | 3 | na | na | na | 6 | 1 | | 3 | |
| Slotman et al., 2017 | T | 8 | 2 | 2 | 1 | na | 2 | na | na | na | 7 | 1 | | 1 | |
| Slotman et al., 2017 | M | 8 | 2 | 2 | 1 | na | 2 | na | na | na | 7 | 1 | | 1 | |
| Snijder et al., 2017 | T | 8 | 2 | 2 | 3 | na | 3 | na | na | na | 10 | 2 | | 3 | |
| Snijder et al., 2017 | M | 8 | 2 | 2 | 3 | na | 3 | na | na | na | 10 | 2 | | 3 | |
| Stronks et al., 2020 | T | 8 | 2 | 3 | 2 | na | 2 | na | na | na | 10 | 2 | | 2 | |
| Stronks et al., 2020 | M | 8 | 2 | 3 | 2 | na | 2 | na | na | na | 10 | 2 | | 2 | |
| Szabo et al., 2020 | M | 9 | 2 | 2 | 1 | na | 2 | na | na | na | 7 | 1 | | 1 | |
| Tagay et al., 2008 | T | 8 | 2 | 3 | 3 | na | 3 | na | na | na | 10 | 2 | | 3 | |
| Ünlu et al., 2014 | T | 10 | 2 | 3 | 2 | na | 2 | na | na | na | 8 | 2 | | 2 | |
| Uslucan, 2005 | T | 12 | 2 | 3 | 1 | na | 3 | na | na | na | 10 | 2 | | 3 | |
| Van der Wurff et al., 2004 | T | 7 | 2 | 2 | 1 | na | 2 | na | na | na | 8 | 2 | | 1 | |
| Van der Wurff et al., 2004 | M | 7 | 2 | 2 | 1 | na | 2 | na | na | na | 8 | 2 | | 1 | |
| Van Dijk et al., 2010 | T | 8 | 2 | 2 | 1 | na | 2 | na | na | na | 7 | 1 | | 1 | |
| Van Dijk et al., 2010 | M | 8 | 2 | 2 | 1 | na | 2 | na | na | na | 7 | 1 | | 1 | |
| Van Tilburg et al., 2019 | T | 9 | 2 | 2 | 1 | na | 2 | na | na | na | 7 | 1 | | 1 | |
| Van Tilburg et al., 2019 | M | 9 | 2 | 2 | 1 | na | 2 | na | na | na | 7 | 1 | | 1 | |

Note. Abbreviations: T = Turkish sample, M = Moroccan sample, na = not applicable; Global quality rating 1 = strong quality (SQ), 2 = moderate quality (MQ), 3 = weak quality (WQ).
